# Low-dose X-ray radiodynamic therapy solely based on gold nanoclusters for efficient treatment of deep hypoxic solid tumors combined with enhanced antitumor immune response

**DOI:** 10.7150/thno.78649

**Published:** 2023-01-22

**Authors:** Shengcang Zhu, Feihong Yan, Lulu Yang, Bingyi Li, Ruxian Xue, Wenwen Yu, Yu Wang, Lu Huang, Lijun Wang, Rongcheng Han, Yuqiang Jiang

**Affiliations:** 1 State Key Laboratory of Molecular Developmental Biology, Institute of Genetics and Developmental Biology, Chinese Academy of Sciences, Beijing 100101, China.; 2 University of Chinese Academy of Sciences, Beijing 100049, China.; 3 Single-Molecule and Nanobiology Laboratory, Department of Biochemistry and Biophysics, School of Basic Medical Sciences, Peking University, Beijing 100191, China.

**Keywords:** gold nanoclusters, radiodynamic therapy, antitumor immune, solid tumor

## Abstract

**Background:** Radiodynamic therapy (RDT) is an emerging novel anti-cancer treatment based on the generation of cytotoxic reactive oxygen species (ROS) at the lesion site following the interaction between low-dose X-ray and a photosensitizer (PS) drug. For a classical RDT, scintillator nanomaterials loaded with traditional PSs are generally involved to generate singlet oxygen (^1^O_2_). However, this scintillator-mediated strategy generally suffers from insufficient energy transfer efficiency and the hypoxic tumor microenvironment, and finally severely impedes the efficacy of RDT.

**Methods:** Gold nanoclusters were irradiated by low dose of X-ray (called RDT) to investigate the production of ROS, killing efficiency of cell level and living body level, antitumor immune mechanism and biosafety.

**Results:** A novel dihydrolipoic acid coated gold nanoclusters (AuNC@DHLA) RDT, without additional scintillator or photosensitizer assisted, has been developed. In contrast to scintillator-mediated strategy, AuNC@DHLA can directly absorb the X-ray and exhibit excellent radiodynamic performance. More importantly, the radiodynamic mechanism of AuNC@DHLA involves electron-transfer mode resulting in O_2_^-•^ and HO•, and excess ROS has been generated even under hypoxic conditions. Highly efficient *in vivo* treatment of solid tumors had been achieved via only single drug administration and low-dose X-ray radiation. Interestingly, enhanced antitumor immune response was involved, which could be effective against tumor recurrence or metastasis. Negligible systemic toxicity was also observed as a consequence of the ultra-small size of AuNC@DHLA and rapid clearance from body after effective treatment.

**Conclusions:** Highly efficient* in vivo* treatment of solid tumors had been achieved, enhanced antitumor immune response and negligible systemic toxicity were observed. Our developed strategy will further promote the cancer therapeutic efficiency under low dose X-ray radiation and hypoxic conditions, and bring hope for clinical cancer treatment.

## Introduction

As a noninvasive technique for the treatment of cancer and other diseases, photodynamic therapy (PDT) is generally involved in the co-localization of light, oxygen, and a photosensitizer (PS) to generate highly cytotoxic reactive oxygen species (ROS) 1. Due to the limited penetration depth of visible light that generally used for most of the available PSs, PDT is now mainly limited to the treatment of superficial and flat lesions 2. Thus, for classical PDT, it is still technically challenging for treatment of deep-seated or solid tumors 3, 4. In consideration of their strong tissue penetrability (8-14 cm) 5, X-rays have been established as an ideal excitation source of activating PSs for the treatment of solid tumors 6. Actually, as early as 2002, acridine orange (AO), a traditional PS, combined with low-dose X-ray radiation of about 1-5 Gy had been proved to produce singlet oxygen (^1^O_2_) 7, a process known as radiodynamic therapy (RDT). Recent publications have demonstrated the significant potential of RDT in tumor treatment 8-11, combing low-dose ionizing radiation with PSs to generate excess ROS for efficient killing of cancer cells.

Gold nanostructure materials possess strong X-ray attenuation capability since they contain high-Z element (Au, Z = 79) and thus they are anticipated to be a very competent candidate as radio-sensitizing materials for RDT. The X-ray absorbance coefficient of gold has been proved to be 100-fold higher than that of normal tissue in the KeV energy range 12, 13. Among the available gold nanostructure materials, gold nanoclusters (AuNCs) have attracted extensive attention due to their ultra-small size, excellent biocompatibility, easy surface functionalization, as well as long tumor retention and fast normal tissue clearance 14-16. Different kinds of AuNCs have been developed and utilized for radiotherapy (RT) as radiosensitizers 17 and PDT as PSs 18. For example, a structurally defined levonorgestrel-gold nanocluster consisting of Au_8_(C_21_H_27_O_2_)_8_ was developed and successfully demonstrated as a nano-radiosensitizer for tumor radiotherapy 17. Recently, the aggregates of conjugations of glutathione-protected gold nanolcusters (AuNC@GSH) and rose bengal (RB) with a size of 68.2 nm were prepared, and highly effective *in vivo* tumor treatment had been achieved upon X-ray radiation 19. These above systems are generally composed by AuNCs and traditional PSs, in which AuNCs as nanoscintillators firstly absorb X-ray, and then transfer energy to nearby, well-matched PSs to produce ROS 20, 21. This scintillator-mediated strategy has been successfully utilized in different kinds of systems and some of them have been unitized for *in vivo* tumor treatment 22. However, when applied for tumors treatments, especially for deep-seated solid tumors, this scintillator-mediated strategy generally suffers from low energy-transfer efficiency, suboptimal therapeutic efficacy, as well as biosafe concerns due to their long-term retention within the body as a result of their large size 23. Besides that, for solid tumors, hypoxia is a common characteristic that severely diminished the therapeutic response of RDT 23, 24. Therefore, ultra-small AuNCs based RDT, which can be directly excited by low-dose X-ray radiation, rapidly excreted from body and exhibit excellent radiodynamic performance under hypoxic conditions for solid tumor treatment, has rarely been reported.

Recently, we reported a dihydrolipoic acid coated gold nanoclusters (AuNC@DHLA) based two-photon PDT with a type I mechanism, and efficient *in vivo* tumor treatment has been achieved 18. Herein, we report a novel and effective RDT system solely based on AuNC@DHLA, without additional nanoscintillator or traditional PSs. In contrast to scintillator-mediated strategy, AuNC@DHLA can directly absorb X-ray to generate excess ROS. More importantly, the radiodynamic mechanism of AuNC@DHLA involves electron-transfer mode (O_2_^-•^ and HO•), not traditional energy-transfer mode, and thus efficient ROS can be generated even under hypoxic conditions. Highly efficient treatment of *in vivo* solid tumors has been achieved with our developed AuNC@DHLA RDT. Interestingly, enhanced antitumor immune response was involved, which could be effective against tumor metastasis. As a consequence of the ultra-small size of AuNC@DHLA and rapid body clearance, negligible systemic toxicity was observed.

## Methods

### Preparation of AuNC@DHLA

Fluorescent AuNCs were prepared by etching nonfluorescent AuNPs. A typical synthesis is described as follows: DHLA (14.0 μL) was added to a methanol solution of HAuCl_4_ (5.0 mL, 5.0 mM) at 0 °C, and the mixture was stirred for 30 min. Subsequently, an aqueous solution of NaBH_4_ (1.25 mL, 0.16 M) was quickly added to the mixture under rapid stirring. After 1 h, the generated gold nanoparticles were collected by adding excess HCL until a brown cloudy solution was obtained. Free DHLA, reducing agents, and smaller nanoparticles were removed by discarding the supernatants after centrifugation (4000 rpm, 10 min). The wet precipitate of gold nanoparticles was then redissolved in ultrapure water (18.2 MΩ^.^cm, 6 mL), and NaOH solution (pH 11.9, 6 mL) was added to obtain a transparent solution. The etching process was initiated by the addition of 21.0 μL of DHLA (adjusted to a pH of 5.6), and the temperature was maintained at 55 °C until the precipitate appeared. Finally, the prepared gold nanoclusters were purified by triple centrifugation filtration using filters with a molecular weight cutoff of 10 kDa to remove impurities. The brownish AuNC@DHLA were resuspended in distilled water and stored at 4 °C for later use.

### Cell lines

Hepa 1-6 cells and human embryonic kidney cells (HEK293) were purchased from National biomedical experimental cell resource bank. Huh-7 cells and B16F10 cells were purchased from Pricella. All cells were cultured at 37 °C and 5% CO_2_ in DMEM medium (Gibco, USA) supplemented with penicillin (100 U/mL), streptomycin (100 mg/L), and 10% fetal bovine serum (FBS, Gibco, USA).

### Fluorescence imaging of intracellular ROS generation

To evaluate intracellular ROS production, Hepa 1-6 cells were grown in a confocal dish (35 mm) supplemented with culture medium to allow 80% confluence in 24 h. After incubating with AuNC@DHLA (200 μg mL^-1^) for 2 h at 37 °C, the cells were rinsed and supplemented with fresh culture medium with 2 μM DCFH-DA at 37 °C for 10 min under darkness. The cells were then irradiated with different X-ray doses of 0, 0.1, 0.5, 3.0, and 6.0 Gy (1.0 Gy min^-1^). The cells were then observed on a Zeiss Axio Observer Z1 optical system. Fluorescence emission of DCFH-DA was collected mainly in the green channel through an emission filter at 500-550 nm when excited by a 488 nm laser.

### Colony formation assay

Hepa 1-6 cells were seeded in a confocal dish (35 mm) at a density of 8 × 10^4^ cells per well and incubated over 24 h for cell attachment. Then, the cells were divided into a control group (without the addition of AuNC@DHLA) and a treatment group (200 μg mL^-1^ AuNC@DHLA). Each group was repeated three times. After 2 h, the cell medium was removed from the wells, and then washed these wells with PBS three times. After treating the cells with different doses of X-ray irradiation (0, 0.25, 0.5, 1.0, and 2.0 Gy), the cells were diluted and seeded into 6-well plates at a density of 2000 cells per well. The cells were visualized by crystal violet staining after culture for 10 days.

### DNA damage detection

Immunofluorescence assay for phosphorylated Ser139 on histone H2AX (γ-H2AX) was performed to evaluate the DNA damage. After incubating with AuNC@DHLA (200 μg mL^-1^) for 2 h, the cells were irradiated with X-ray (1.0 Gy) and then fixed with 4% paraformaldehyde for 15 min, permeated with 0.1% Triton X-100 for 15 min, and blocked with 1% bovine serum albumin for 60 min. Afterwards, the cells were incubated with γ-H2AX monoclonal antibody (ThermoFisher, USA) overnight at 4 °C, Alexa Fluor^®^555 goat anti-mouse IgG for 60 min at room temperature, and the nuclei were stained with Hoechst 33342. The cells were then observed on a Zeiss LSM 780 NLO optical system.

### Apoptosis assay

An Annexin V-FITC and DAPI (Solarbio) were used to detect the apoptotic/necrotic cells. After incubating with AuNC@DHLA at 200 μg mL^-1^ for 2 h, the cells were irradiated with X-ray (1.0 Gy). After culture for 1 day, the cells stained with Annexin V-FITC and DAPI according to the manufacturer's protocol. Finally, cells were subjected to flow cytometry (BD FACSAria Fusion cell sorter) and analyzed with FlowJo (V10.6.2).

### Xenograft model

This study was approved by Institutional Animal Care and Use Committee, Institute of Genetics and Developmental Biology, Chinese Academy of Sciences. Four-week-old C57BL/6j mice were purchased from Beijing Vital River Laboratory Animal Technology Co., Ltd. All mice were maintained in a specific pathogen-free house at room temperature with a standard 12 h light/dark cycle. The mice were allowed free access to water and food in the form of a standard pellet diet. Hepa 1-6 cells (2 × 10^6^ cells in 0.1 mL of PBS) were injected subcutaneously into the right flank of C57BL/6j mice to establish a model of tumor-bearing mice. Tumor growth was monitored by periodic Vernier caliper measurements. The tumor volume was calculated with the formula: tumor volume = (width^2^ × length)/2.

### *In vivo* antitumor effects

When the tumors reached a volume of about 100 mm^3^, the mice were randomized into four groups: control, X-ray (only), AuNC, and RDT (AuNC@DHLA + X-ray). The mice in the control group were intratumorally injected with PBS (pH 7.4, 100 μL) on day 0. The mice in the AuNC group were intratumorally injected with only AuNC@DHLA (6.1 mg kg^-1^, 100 μL). The mice in the X-ray group were anesthetized, and their tumor sites were irradiated by 0.25 Gy dose of X-ray (1.0 Gy min^-1^) on an X-ray irradiator (Rod Source technologies Asia Limited, RS2000, America). The mice in the RDT group were intratumorally injected with AuNC@DHLA (6.1 mg kg^-1^, 100 μL) on day 0, followed by only once X-ray radiation (0.25 Gy, 1.0 Gy min^-1^) on an X-ray irradiator (Rod Source technologies Asia Limited, RS2000, America).

### Tumor challenge test

Tumors were established by subcutaneous inoculation of Hepa 1-6 cell suspension (2 × 10^6^ cells per mouse) into the right flank region. When the tumors reached 100 mm^3^ in volume, AuNC@DHLA was intratumorally injected followed by X-ray irradiation at a dose of 1.0 Gy fraction^-1^ (160 kVp, 25 mA, 0.3 mm Cu filter) for a total of 6 fractions on consecutive days. On day 28, mice were challenged by subcutaneous injection of Hepa 1-6 cell suspension (2 × 10^6^ cells per mouse) into the left flanks. The same numbers of Hepa 1-6 cells were inoculated on tumor-free mice as a control. On day 71, the four survived mice were challenged by subcutaneous injection of B16F10 cell suspension (5 × 10^6^ cells per mouse) into the back of neck. The same numbers of B16F10 cells were inoculated in tumor-free mice as a control. The tumor sizes were measured with a vernier caliper every other day and the tumor volumes were calculated by (width^2^ × length)/2.

### Tumor-infiltrating immune cells

Tumor tissues were harvested from mice in different groups, cut up and next removed red cells by red blood cell lysis buffer. Those remaining cells were labeled by anti-FOXP3-Alexa Fluor®647 (Biolegend, Clone: MF-14, Catalog: 126408), anti-MHC II-APC (Dogesce, Clone: M5/114.15.2, Catalog: 17-5321-81), anti-CD11c-eFluor 450 (eBioscience, Clone: N418, Catalog: 48-0114-82), anti-F4/80-Brilliant Violet 711™ (eBioscience, Clone: BM8, Catalog: 123147), anti-CD45R (B220)-PE-Cyanine7 (eBioscience, Clone: RA3-6B2, Catalog: 25-0452-82), anti-NK1.1-PE (eBioscience, Clone: PK136, Catalog: 12-5941-82), anti-CD4-eFluor 506 (Thermofisher, Clone: RM4-5, Catalog: 69-0042-82), anti-CD8a-APC-Cyanine7 (Biolegend, Clone: 53-6.7, Catalog: 100714), anti-CD3-PerCP-eFluor 710 (eBioscience, Clone: 17A2, Catalog: 46-0032-82), anti-CD45-FITC (eBioscience, Clone: 30-F11, Catalog: 11-0451-82) antibodies according to the manufacturer's protocols. All these antibodies used in our experiments were diluted for 100 times. T cells were labelled by CD45^+^ CD3^+^. CD4^+^ T cells were labelled by CD45^+^ CD3^+^ CD4^+^. CD8^+^ T cells were labelled by CD45^+^ CD3^+^ CD8ɑ^+^. NK cells were labelled by CD45^+^ CD3^-^ NK1.1^+^. B cells were labelled by CD45^+^ CD3^-^ B220^+^. DCs were labelled by CD45^+^ CD3^-^ B220^-^ F4/80^-^ CD11c^+^ MHC II^+^. Macrophages were labelled by CD45^+^ CD3^-^ B220^-^ F4/80^+^. Treg cells were labelled by CD45^+^ CD3^+^ CD4^+^ FOXP3^+^.

### Tumor-infiltrating PD-1 on CD8^+^ T cells and CTLA-4 on CD4^+^ T cells

Tumor tissues were harvested from mice in different groups, cut up, and next removed red cells by red blood cell lysis buffer. Those remaining cells were labeled by anti-CD279 (PD-1)-PE (eBioscience, Clone: J43, Catalog: 12-9985-82), anti-CD152 (CTLA-4)-PE-eFluor610 (eBioscience, Clone: UC10-4B9, Catalog: 61-1522-8), CD8a-APC-Cyanine7 (Biolegend, Clone: 53-6.7, Catalog: 100714), anti-CD4-eFluor 506 (Thermofisher, Clone: RM4-5, Catalog: 69-0042-82), anti-CD3-PerCP-eFluor 710 (eBioscience, Clone: 17A2, Catalog: 46-0032-82), anti-CD45-FITC (eBioscience, Clone: 30-F11, Catalog: 11-0451-82) antibodies according to the manufacturer's protocols. All these antibodies used in our experiments were diluted for 100 times. PD-1 was labelled by CD45^+^ CD3^+^ CD8ɑ^+^ PD-1^+^, CTLA-4 was labelled by CD45^+^ CD3^+^ CD4^+^ CTLA-4^+^.

### T central memory cells

Spleen tissues were harvested from mice in different groups, crushed, and next removed red cells by red blood cell lysis buffer. Those remaining cells were labeled by anti-CD44-APC (eBioscience, Clone: IM7, Catalog: 17-0441-82), anti-CCR7-PE (abcam, Clone: 4B12, Catalog: ab95669), anti-CD62L-APC (abcam, Clone: MEL-14, Catalog: ab41459), anti-CD8a-APC-Cyanine7 (Biolegend, Clone: 53-6.7, Catalog: 100714), anti-CD3-PerCP-eFluor 710 (eBioscience, Clone: 17A2, Catalog: 46-0032-82), anti-CD45-FITC (eBioscience, Clone: 30-F11, Catalog: 11-0451-82) antibodies according to the manufacturer's protocols. All these antibodies used in our experiments were diluted for 100 times. T central memory cells were labelled by CD45^+^ CD3^+^ CD8^+^ CD44^+^ CCR7^+^ CD62L^+^.

T central memory cells in peripheral blood were detected using the same method described above, excepting for the step of being crushed.

### *In vivo* CT imaging

Mice bearing Hepa 1-6 cell tumors were intratumorally injected with AuNC@DHLA (10 mg mL^-1^, 100 µL). The mice were anesthetized under isoflurane and scanned by a home-made Computed Tomography system (ZCB-100) before intratumoral injection of gold nanoclusters and at 0.5 h, 2 h, 8 h, 12 h, 24 h, 36 h, and 48 h after administration of the clusters. The CT scanning was performed at a tube voltage of 90 kV, current of 2.5 mA, and gantry rotation time of 18 s. CT images were reconstructed and the Hounsfield unit (HU) was quantified at the tumor areas.

### Biodistribution

At 48 h post injection of AuNC@DHLA, the mice were sacrificed after the last CT scanning. Hepa 1-6 tumors and organs, including liver, spleen, heart, kidneys, stomach, lung, intestine, blood and brain, were discretized, weighed, immersed in aqua regia and trypsinized at 60 °C for 1 day. When all the tissues were completely trypsinized, the aqua regia solution was diluted with DI water and then measured by ICP-MS to determine the Au content.

### *In vivo* toxicity

Mice were weighted and assessed for behavioral changes. On the 20^th^ day after treatment, all mice were sacrificed, and their blood and organs were collected for hematology, biochemistry and toxicological investigation. The blood was drawn for hematology analysis and serum biochemistry analysis. During necropsy, liver, kidney, spleen, heart, lung, and tumor were collected and then fixed in 4% neutral buffered paraformaldehyde, processed into paraffin, sectioned into 5 μm slices, and mounted onto glass slides. After H&E staining, images were acquired with a Pannoramic MIDI digital slice scanner (3D HISTECH, Hungary).

### Statistical analysis

Quantitative results are presented as mean ± standard deviation. Differences between groups were evaluated using an unpaired One or two-tailed Student's t-test. Differences were considered statistically significant at *p <* 0.05. (*P < 0.05; **P < 0.01; ***P < 0.001).

## Results and Discussion

### Size characterization and X-ray absorption properties

AuNC@DHLA was prepared by chemical etching method, with the characteristics of small size (1.7 ± 0.4 nm, determined by TEM, Figure S1A-B) and the same spectral profile as our previous report (Figure S1C) 18. The hydrodynamic diameter of AuNC@DHLA (~ 4.4 nm, Figure S1D), determined by dynamic light scattering (DLS), was bigger than the size determined by TEM because of DHLA anchored on the surface of the gold core nanoparticles and the thickness of the electrical double layer (solvation shell) 25. The small size of AuNC@DHLA is beneficial for rapid body clearance via renal excretion 26, and suppression of the reticuloendothelial system (RES) uptake in unintended organs, such as the liver and spleen 27, as well as any potential AuNCs induced organ toxicity.

**Figure 1:**
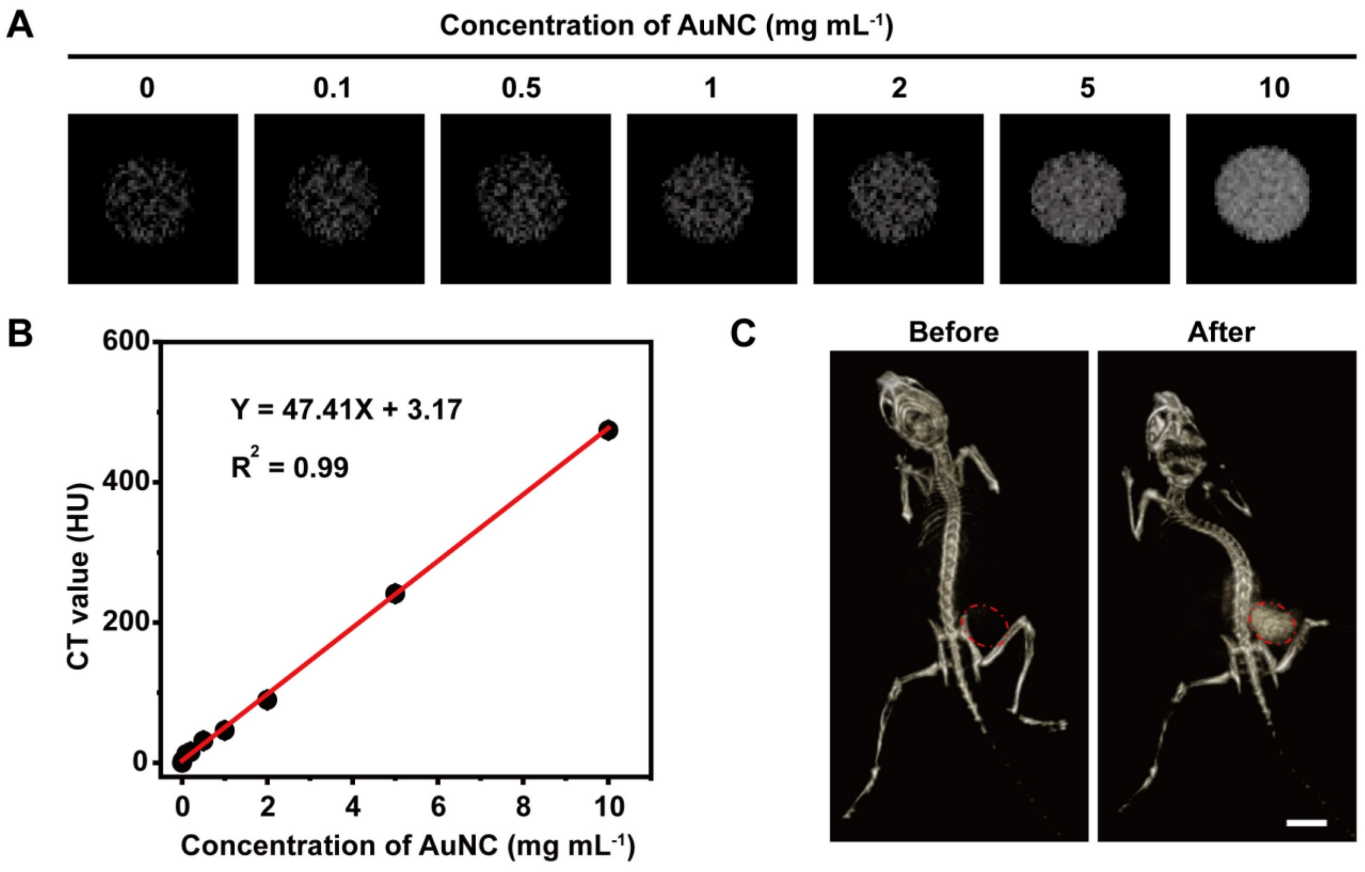
** (A)** *In vitro* CT images of AuNC@DHLA with various concentrations. **(B)** CT values in Hounsfeild unit (HU) of AuNC@DHLA as a function of concentration. **(C)** CT images of a tumor bearing C57BL/6j mouse before and after intratumor injection with AuNC@DHLA (10.0 mg mL^-1^, 100 μL) at 8 h. The CT contrast was obviously enhanced in the mouse tumor (red dashed circle). Scale bar: 1 cm.

Then the X-ray absorption ability of AuNC@DHLA was assessed with CT imaging. The parameters, such as tube voltage and tube current, for CT imaging of AuNC@DHLA were carefully optimized to obtain high quality CT images (Figure S2A-B). CT images of AuNC@DHLA aqueous solution at different concentrations were demonstrated in Figure 1A, and the CT signals linearly increased along with the concentration of AuNC@DHLA (Figure 1B). The slope of the HU value for AuNC@DHLA was about 47.41 HU L g^-1^, which was much higher than that of iopromide (15.9 HU L g^-1^) 28, a commercial iodine-based CT contrast agent used in the clinic. The strong X-ray absorption ability of AuNC@DHLA was further confirmed by *in vivo* CT imaging. C57BL/6j mice bearing Hepa 1-6 tumors were intratumorally injected with AuNC@DHLA and then imaged by a home-made ZCB-100 instrument (Figure 1C). Strong tumor contrast was observed in the CT image, with the CT gray value dramatically increased from 6.19 ± 5.66 before injection to 131.78 ± 13.29 after injection.

### The radiodynamic properties

**Figure 2:**
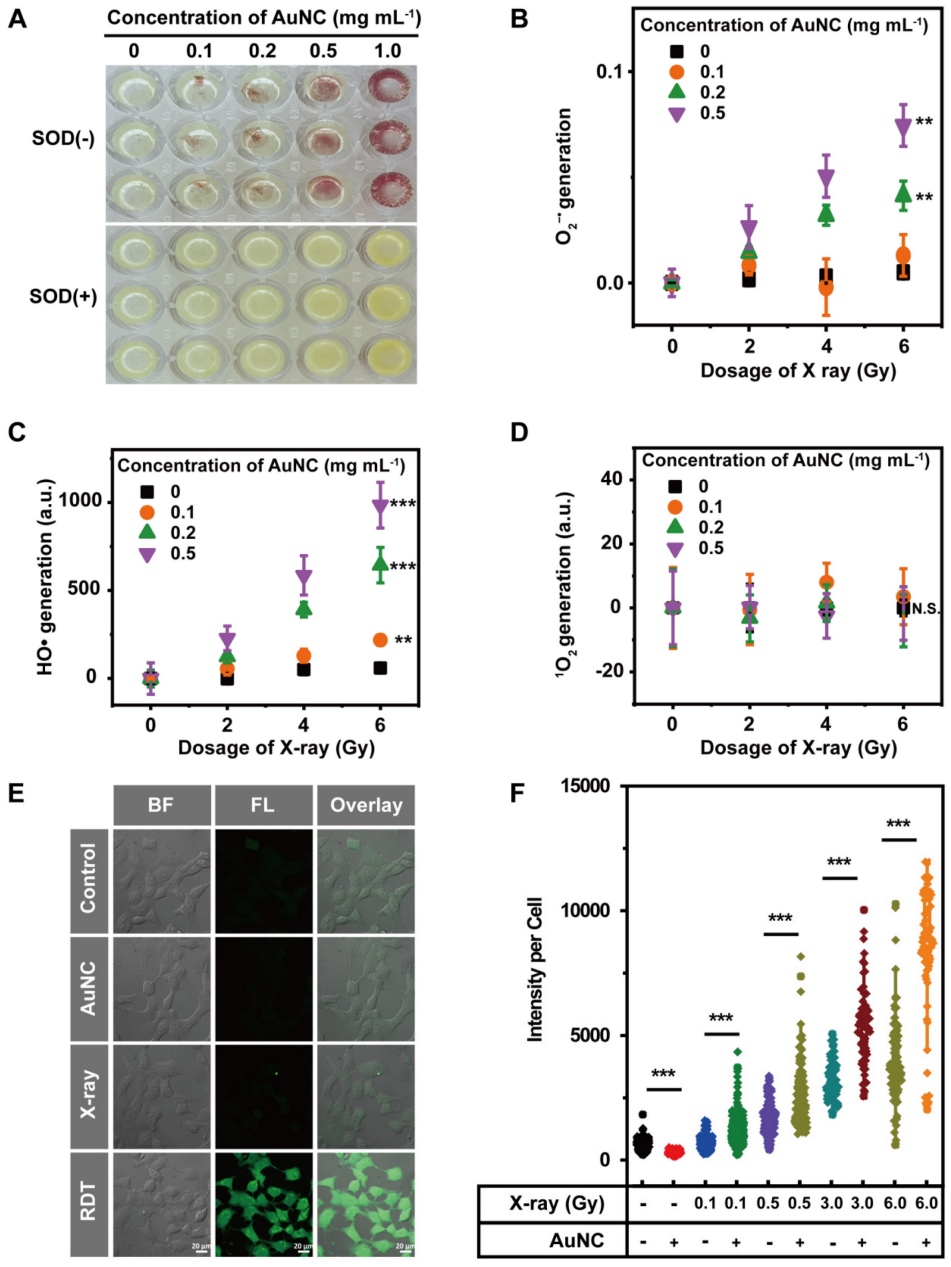
** (A)** The insoluble purple formazan product generated in the AuNC@DHLA aqueous solution when upon X-ray radiation (6.0 Gy). Moreover, more formazan could be observed with increasing the concentration of AuNC@DHLA. In the presence of superoxide dismutase (SOD), a scavenger of O_2_^-•^, these insoluble formazans could not be observed. **(B)** The amount of O_2_^-•^ as a function of the dosage of X-ray. Increasing the dosage of X-ray generally results in more amount of O_2_^-•^. Besides that, under the same X-ray radiation conditions, more AuNC@DHLA generally produce more amount of O_2_^-•^. Statistical analysis was performed by two-tailed t-test (**p < 0.01). **(C)** Generation of HO• during X-ray radiation in the presence of AuNC@DHLA was analyzed using 3′-(*p*-aminophenyl) fluorescein (APF) as the HO• trap. The amount of generated HO• increased with increasing the dosage of X-ray. Statistical analysis was performed by two-tailed t-test (**p < 0.01, ***p < 0.001). **(D)** Generation of ^1^O_2_ from AuNC@DHLA upon X-ray radiation was analyzed with singlet oxygen sensor green (SOSG) assay. No apparent signal of ^1^O_2_ could be detected. Statistical analysis was performed by two-tailed t-test (N.S. *p* > 0.05). **(E)** Images of Hepa 1-6 cells stained with 2′,7′-dichlorodihydrofluorescein diacetate (DCFH-DA) after treatment with AuNC@DHLA and X-ray (0.5 Gy). For comparison, other controls, including treatment with AuNC@DHLA (200 μg mL^-1^) alone (without X-ray) and X-ray alone, are also demonstrated. The blank control group of cells received neither AuNC@DHLA nor X-ray. BF: bright field; FL: fluorescence after staining with 2′, 7′-dichlorodihydrofluorescein diacetate (DCFH-DA). **(F)** Corresponding intracellular fluorescence intensity of Hepa 1-6 cells (N ≥ 50). The concentration of AuNC was 200 μg mL^-1^. The box plot showed data points, and one point corresponded to the intensity per cell. Statistical analysis was performed by two-tailed t-test (***p < 0.001).

The radiodynamic properties of AuNC@DHLA were further investigated upon X-ray radiation. Three mainly ROS, superoxide anion (O_2_^-•^), hydroxyl radical (HO•), and singlet oxygen (^1^O_2_), have been measured with Nitro Blue Tetrazolium (NBT) assay, 3'-(p-aminophenyl) fluorescein (APF) assay, and singlet oxygen sensor green (SOSG) assay, respectively. For NBT assay, when O_2_^-•^ generated, brown and insoluble products (formazan) would appear. As shown in Figure 2A, brown and insoluble products could be observed with AuNC@DHLA upon X-ray radiation and these insoluble products disappeared after the addition of superoxide dismutase (SOD), a scavenger of O_2_^-•^ 29, 30. Further quantitative analysis indicated that more O_2_^-•^ could be generated with increasing the dose of X-ray or the amount of AuNC@DHLA (Figure 2B). These above results proved that O_2_^-•^ can be generated from AuNC@DHLA upon X-ray radiation. Besides O_2_^-•^, HO• could also be detected from AuNC@DHLA upon X-ray radiation via APF assay. As shown in Figure 2C, the amount of HO• increased with the dosage of X-ray and decreased with the addition of tert-butyl alcohol (Figure S3A), an inhibitor of HO•. Similar results can also be obtained for AuNC@DHLA upon one-photon/two-photon excitation via electron spin resonance (ESR) spectroscopy, NBT assay, APF assay, respectively (Figure S3B-D). However, for our AuNC@DHLA RDT system, even when radiated by X-ray and two-photon excitation, no apparent signal of ^1^O_2_ was detected (Figure 2D and Figure S3E). All these above results indicated that the radiodynamic mechanism of AuNC@DHLA mainly involves electron-transfer mode (Type I process, generating O_2_^-•^ and HO•), not traditional energy-transfer mode (Type II process, generating ^1^O_2_) 31.

As for the mechanism behind the efficient Type I process, we infer that the complex energy level structure of AuNC and the long triplet lifetime (~μs, data not shown) are highly plausibly involved. Upon X-ray irradiation, the excited singlet state (^1^AuNC@DHLA*) generates and is followed by efficient intersystem crossing (ISC) to an excited triplet state (^3^AuNC@DHLA*). In other words, the triplet formation originates from an effective excited-state relaxation from the initially populated singlet (S_1_) to triplet (T_1_) states via an intermediate triplet (T_2_) state. The low reduction potential and long lifetime of the T_1_ state facilitate the efficient generation of ROS (O_2_^-•^, HO•) by charge transfer between photosensitizer (AuNC@DHLA) and substrate molecules. The negligible ^1^O_2_ could be ascribed to the small energy gap of T_1_-S_0_ that is smaller than that between ^3^O_2_ and ^1^O_2_, just as described in the literature 32. Meanwhile, previous studies have proved that thiol compounds could generate superoxide via autoxidation reaction 33, 34, and thus excess thiols or free thiols liberated from the Au surface may also participate in oxidations catalyzed by thiol-protected AuNCs to generate final ROS.

In aqueous solution the superoxide anion O_2_^-•^ exists in an acid-base equilibrium with its protonated form, the perhydroxyl radical (HO2) (Equation 1).

HO_2_ ↔ H^+^ + O_2_^-•^ (1)

The protonated perhydroxyl radical (HO_2_) is generally much more reactive toward organic molecules than the superoxide anion form 35. On the other hand, the half-life of O_2_^-•^ is very long and highly pH-dependent, with a value of 0.5 s at pH 6.5 and 50 s at pH 8.5 36. Under physiological conditions, superoxide anion can be converted to hydrogen peroxide (H_2_O_2_), and sequentially, a hydroxyl radical (HO•) is produced via a Fenton reaction 37, 38. For cells, HO• is more destructive and harmful and is likely to be produced if its precursor is not scavenged by antioxidants in time 36, 38. Based on the efficient generation of O_2_^-•^ and HO•, high radiodynamic performance and efficient killing cancer cells can be expected for AuNC@DHLA RDT.

An intracellular ROS assay was used to further evaluate the feasibility of AuNC@DHLA-based radiodynamic therapy (RDT). As shown in Figure 2E, more ROS, indicated by green fluorescence, was observed in the RDT (AuNC@DHLA + X-ray) group than that of other groups. The radiodynamic ability of AuNC@DHLA was further confirmed under various doses of X-ray, as shown in Figure 2F. The strong ROS generation ability endows AuNC@DHLA to be one potential anticancer agent for RDT.

### Cellular radiodynamic performance

**Figure 3:**
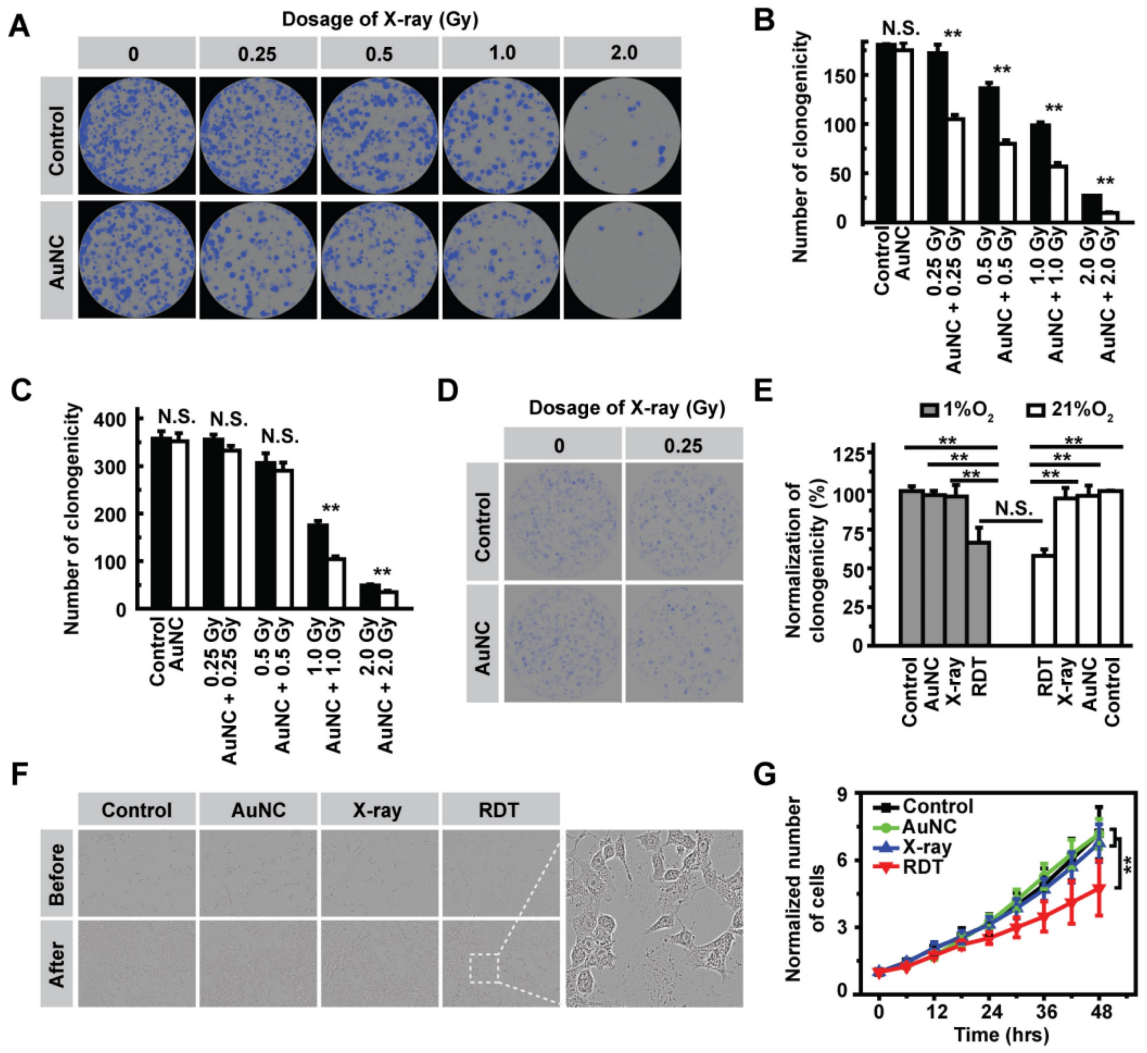
** (A)** Representative images of the colony formation assay and **(B)** Statistical results of the surviving fraction of Hepa 1-6 cells with different treatments in normoxic condition (21% O_2_). Statistical analysis was performed by two-tailed t-test (**p < 0.01). **(C)** Statistical results of the surviving fraction of 293T cells with different treatments in normoxic condition (21% O_2_). Statistical analysis was performed by two-tailed t-test (**p < 0.01). **(D)** Representative images of the colony formation assay of Hepa 1-6 cells in hypoxic condition (1% O_2_). **(E)** Statistical results of the surviving fraction of Hepa 1-6 cells under 1% O_2_ and 21% O_2_ condition. Statistical analysis was performed by two-tailed t-test (**p < 0.01). **(F)** Representative bright field imaging of Hepa 1-6 cells before and after RDT treatment at 48 h. Inset: A magnified region of the cancer cells after AuNC@DHLA + X-ray (1.0 Gy) treatment. Abnormal cell morphology could be obviously observed. **(G)** The relative number of cells at different time points after treatments. Statistical analysis was performed by two-tailed t-test (**p < 0.01). The AuNC group: 100 μg mL^-1^; The X-ray group: 1.0 Gy; The RDT group: 100 μg mL^-1^ AuNC@DHLA + 1.0 Gy X-ray.

Encouraged by the strong ROS generation ability of AuNC@DHLA upon X-ray radiation, we next performed *in vitro* experiments to explore the efficacy of AuNC@DHLA RDT in killing cancer cells. Prior to *in vitro* efficacy assays, the dark cytotoxicity of AuNC@DHLA was firstly assessed by real time bright field imaging tracking. As shown in Figure S4A-B, without X-ray radiation, no apparent cytotoxic effect was observed in Hepa 1-6 cells even when the concentration of AuNC@DHLA reached as high as 1.0 mg mL^-1^ at the 24-hour time point. The superior biocompatibility of AuNC@DHLA was further confirmed with Huh-7 and 293T cells via MTT method (Figure S4C-D). The cellular RDT efficacy of AuNC@DHLA was assessed by the cell colony formation assay, in which the lethal effects on cancer cells increased as the cell colonies decreased, and *vice versa*. As shown in Figure 3A-B and Table S1, under normoxic conditions (21% O_2_), AuNC@DHLA combined with X-ray radiation could more efficiently kill cancer cells than the X-ray group or AuNC@DHLA group. In addition, the cellular RDT efficacy was proportional to the dose of X-ray. As shown in Figure S5A, the number of cell death in the RDT group, measured by propidium iodide (PI), was obviously more than that of other groups. Similar results were also observed in 293T and Huh-7 cells (Figure 3C, Figure S5B-C). Importantly, under hypoxic conditions (1% O_2_), the efficacy of AuNC@DHLA RDT still reached up to 33.5 ± 9.3%, slightly lower than that of under normoxic condition (41.9 ± 2.4%), as shown in Figure 3D-E. Further statistical analysis indicated that there was no significant difference in cell killing efficiency between normoxic condition and hypoxic condition (*p* = 0.2723, Figure 3E), which can be mainly ascribed to the electron-transfer mode of AuNC@DHLA based RDT. As discussed above, in contrast to ^1^O_2_, the characteristic of O_2_^-•^ and HO• generated in our developed AuNC@DHLA RDT has relative longer lifetime and much higher reactivity, and thus finally to be greatly beneficial to the superior efficacy.

For solid tumors with a diameter of > 1 mm 39, insufficient oxygenation (hypoxia) and acidic pH (acidosis) are characteristic abnormalities of the tumor microenvironment (TME) 40. This hypoxia TME can activate angiogenesis, increase risk of tumor metastasis, as well as suppress anti-tumor immunity and hamper the therapeutic response 41, 42. The fact that our developed AuNC@DHLA RDT exhibited excellent cell killing efficiency even under abnormal TME is a great advantage for efficient solid tumor treatment.

The *in vitro* efficacy of AuNC@DHLA RDT was also assessed with the inhibition of cell division. In contrast to long-term cell colony formation, AuNC@DHLA RDT could also inhibit cell division on a short time scale (< 48 h), as shown in Figure 3F-G. Apparent abnormal cell morphology could be observed (Figure 3F). And there was significant difference between the X-ray group (1.0 Gy) and the RDT group (AuNC + 1.0 Gy), as shown in Figure 3G. Compared with the RDT group (AuNC + 0.5 Gy) in Figure S6A, a sharp increase of inhibition ratio (from 13.56% to 34.11%) was observed when the cells were treated with RDT treatment (AuNC + 1.0 Gy). Similar inhibition results were also obtained under different conditions (Figure S6B-E). Collectively, these above results indicated that AuNC@DHLA was an excellent photosensitizer for RDT.

**Figure 4:**
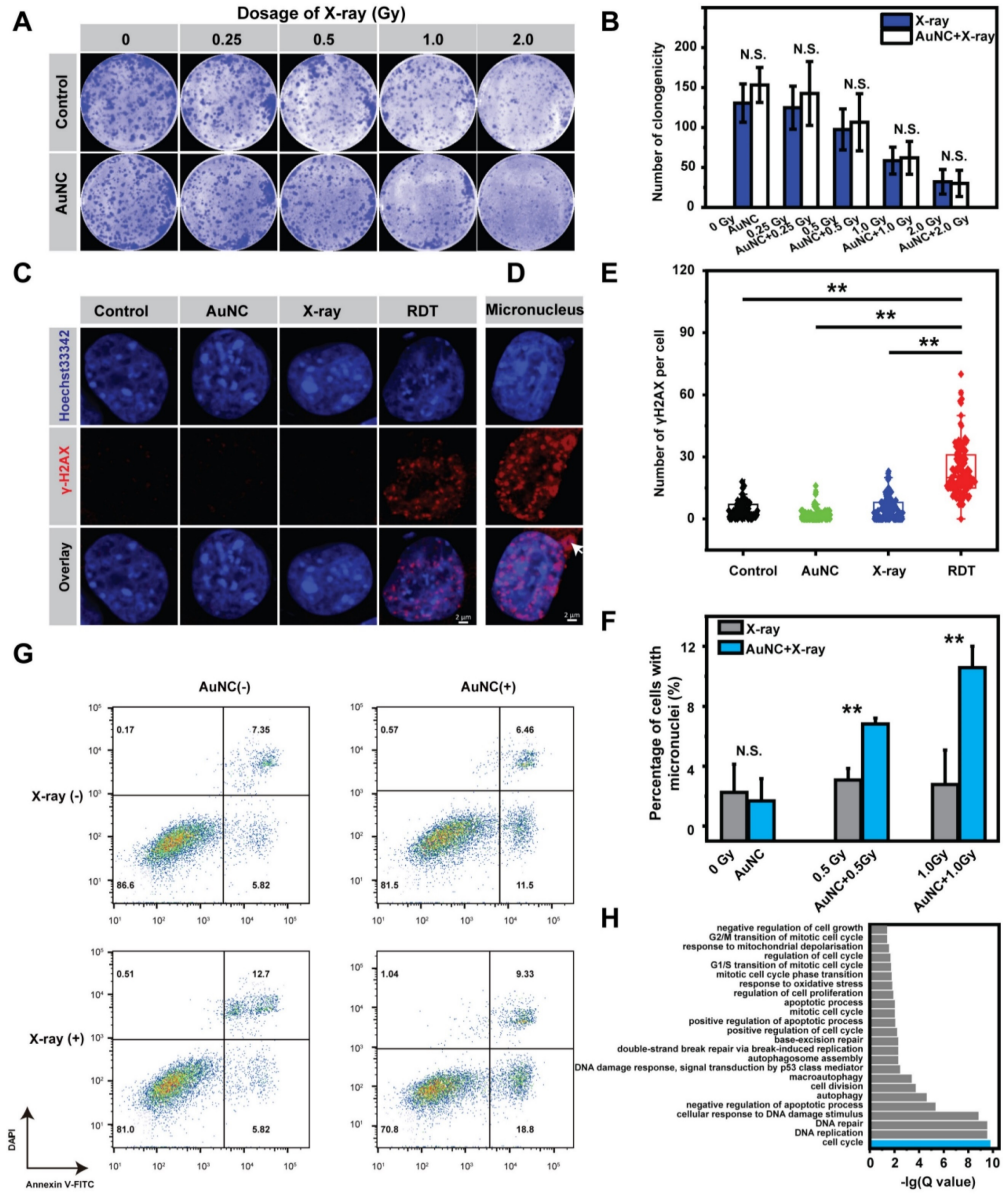
** (A)** Representative images of the colony formation assay and **(B)** Statistical results of the surviving fraction of Hepa 1-6 cells with different treatments in the presence of ROS inhibitor vitamin C. The concentration of AuNC@DHLA was 200 μg mL^-1^. Statistical analysis was performed by two-tailed t-test (N.S. *p* > 0.05). **(C)** DNA damage as measured by Alexa Fluor^®^ 555 second antibody (red), γ-H2AX monoclonal antibody and Hoechst 33342 (blue) for visualizing DNA fragmentation and nucleus respectively in Hepa 1-6 cells with and without AuNC@DHLA (200 μg mL^-1^) under X-ray radiation (1.0 Gy). **(D)** Representative images of micronucleus, indicated by white arrow. **(E)** The corresponding statistical result of γ-H2AX per cell (N ≥ 100) induced by different treatments as indicated. The concentration of AuNC@DHLA was 200 μg mL^-1^. Statistical analysis was performed by two-tailed t-test (**p < 0.01). **(F)** The percentage of Hepa 1-6 cells with micronucleus after receiving different treatments (control group, AuNC@DHLA group, X-ray group (0.5 Gy or 1.0 Gy), AuNC@DHLA + X-ray group (0.5 Gy or 1.0 Gy). The concentration of AuNC@DHLA was 200 μg mL^-1^. Statistical analysis was performed by two-tailed t-test (**p < 0.01). **(G)** Apoptosis results of Hepa 1-6 cells treated without (control) and with AuNC@DHLA (200 μg mL^-1^) before and after X-ray radiation (1.0 Gy). **(H)** Bar plot of biological function enriched at FDR < 5% for AuNC@DHLA based radiodynamic therapy. N = 3 independent samples per group. FDR, false discovery rate.

The cellular mechanism of AuNC@DHLA RDT was further investigated. The effect of ROS on the killing efficiency of Hepa 1-6 cells was first assessed by adding ascorbic acid (Vitamin C) (Figure S7A-B), a scavenger of ROS 43. As shown in Figure 4A-B, in the presence of Vitamin C, there was no difference of the killing efficiency between the RDT group and the X-ray group, which confirmed that ROS did induce the radiodynamic effect. Subsequently, immunofluorescence assay for phosphorylated Ser139 on histone H2AX (γ-H2AX) was performed to evaluate the degree of DNA double-strand breaks (DSBs) 44, 45. As shown in Figure 4C, without X-ray radiation, negligible DNA damage was observed for the cells pretreated with AuNC@DHLA. In contrast, upon low-dosage X-ray (~ 1.0 Gy) radiation, these AuNC@DHLA treated cells exhibited a significant increase of DNA damage (Figure 4C-E), and micronucleus (as indicated with white arrow in Figure 4D) could be also observed. The mean number of γ-H2AX in the RDT group was nearly five times higher than that in the control group. Similar DNA damage with different doses of X-ray was also observed (Figure S8A-B).

These above results proved that AuNC@DHLA RDT did induce cellular DNA damage. These DNA damages can be accumulated and promote DNA repair and arrest the cell cycle, or even trigger apoptosis 46-48. Non-homologous end joining (NHEJ) is the major pathway for DSBs repair in mammalian cells, repairing DSBs in all cell cycle phases 49, 50. As shown in Figure S9, for the RDT group, cells were arrested on the G1/S checkpoint. And the percentage of apoptotic cells slightly increased from 13.17 % to 18.52% after X-ray radiation treatment alone (Figure 4G). In contrast, a sharp increase of apoptosis ratio from 13.17 % to 28.13% was observed when the cells were treated with both AuNC@DHLA and X-ray radiation (Figure 4G). These above results indicate that AuNC@DHLA RDT could induce DNA damage, cell cycle arrest (G1/S checkpoint) and apoptosis. Further mRNA sequencing analysis (Figure 4H) confirmed this above conclusion and also indicated that RDT induced response to oxidative stress and mitochondrial depolarization, negative regulation of cell growth and autophagy.

As above mentioned, micronuclei were observed in the RDT group, which contain damaged DNA surrounded by nuclear envelope and occur after mis-segregation of DNA during cell division. The percentage of cells with micronucleus in the RDT group was significantly higher than the X-ray group (**p < 0.01, Figure 4F). In addition, the percentage of cells with micronuclei in the RDT group increased with increasing X-ray dose or extending the time scale after treatment (Figure S10A-B). Importantly, these micronuclei change can be not only involved in genomic instability, but also in eliciting an immune response 51-53.

### *In vivo* antitumor effects

**Figure 5:**
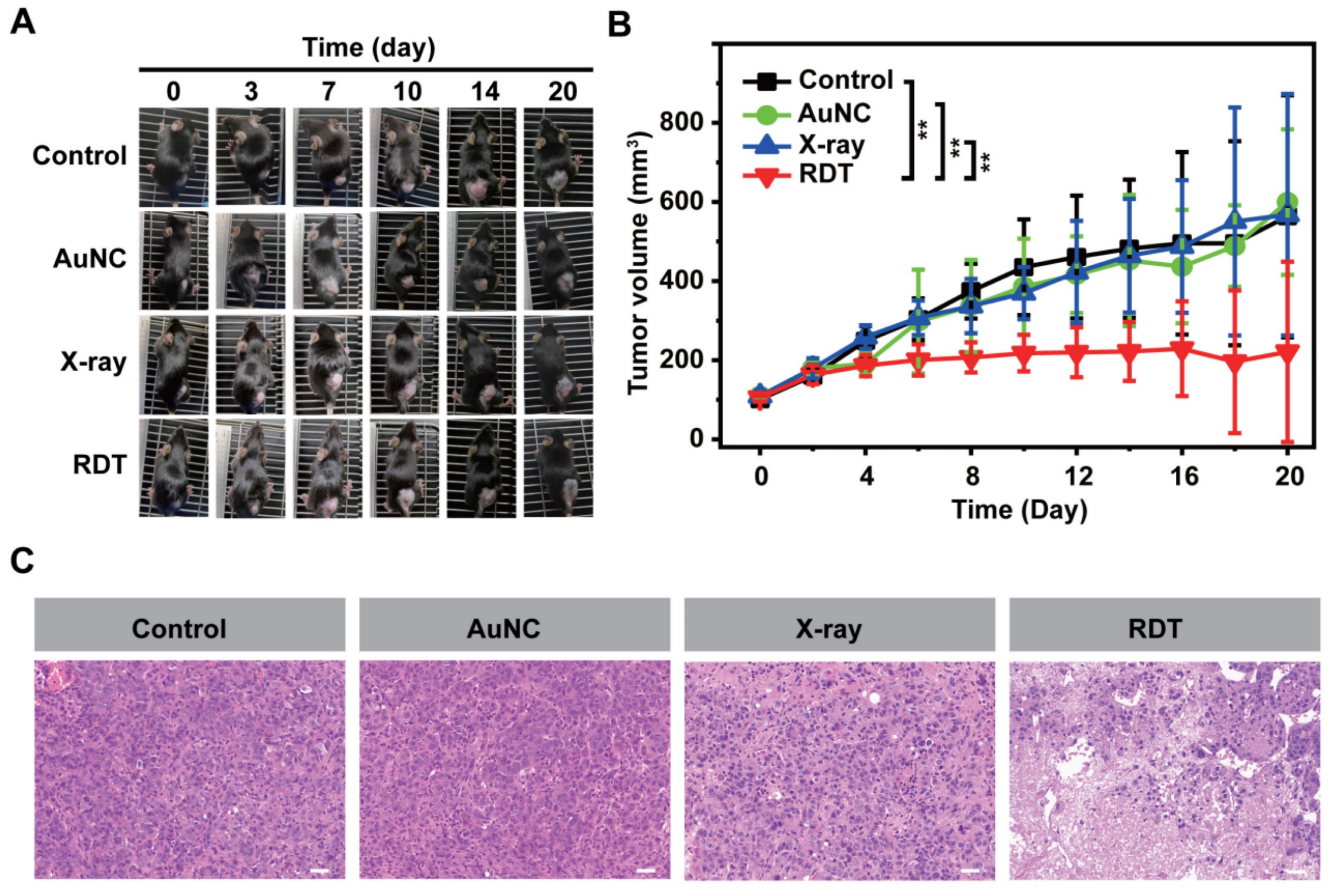
***In vivo* AuNC@DHLA-based radiodynamic therapy.** The day of injection was designated as day 0.** (A)** Photo images of Hepa 1-6 tumors in mice after different treatment. These mice injected only with PBS were designated as the control group. These mice irradiated with X-ray at dose of 0.25 Gy (1.0 Gy min^-1^), without AuNC@DHLA, were designated as the X-ray group. These mice injected with AuNC@DHLA (6.1 mg kg^-1^), without X-ray radiation, were designated as the AuNC group. These mice injected with AuNC@DHLA (6.1 mg kg^-1^) and irradiated with X-ray at dose of 0.25 Gy (1.0 Gy min^-1^) were designated as the RDT group. **(B)** Tumor growth curve of Hepa 1-6 tumor-bearing C57BL/6j mice from different groups within 20 days. Data are represented as mean ± SD (4 mice per group). Data were analyzed by two-tailed t-test (**p < 0.01). **(C)** Slices of H&E staining of tumors with different treatment.

To assess the *in vivo* efficacy of AuNC@DHLA RDT, xenograft models of hepatocellular carcinoma were used and randomly divided into four groups: 1) the control group, 2) the AuNC group with only AuNC@DHLA (6.1 mg kg^-1^), 3) the X-ray group with a dose of 0.25 Gy, 4) the RDT group with AuNC@DHLA (6.1 mg kg^-1^) and X-ray (0.25 Gy). Mice in the RDT group were intratumorally injected with AuNC@DHLA on day 0, followed by X-ray radiation only once after injection. As shown in Figure 5A-B and Table S2, AuNC@DHLA RDT exhibited an obvious inhibition of tumor growth, whose volumes of tumor were much smaller than those of other groups (**p < 0.01), confirming an effective treatment of tumors. No significant differences were observed among the control group, AuNC group and X-ray group. Further tumor sections stained with hematoxylin and eosin (H&E) indicated that the majority of tumor cells lost their normal morphology after RDT treatment (Figure 5C). In contrast, other two control groups (AuNC group and X-ray group) showed normal cell morphologies similar to the untreated control group. These results indicate that our developed AuNC@DHLA RDT could be highly efficient for *in vivo* treatment of solid tumors.

### Antitumor immune response

**Figure 6:**
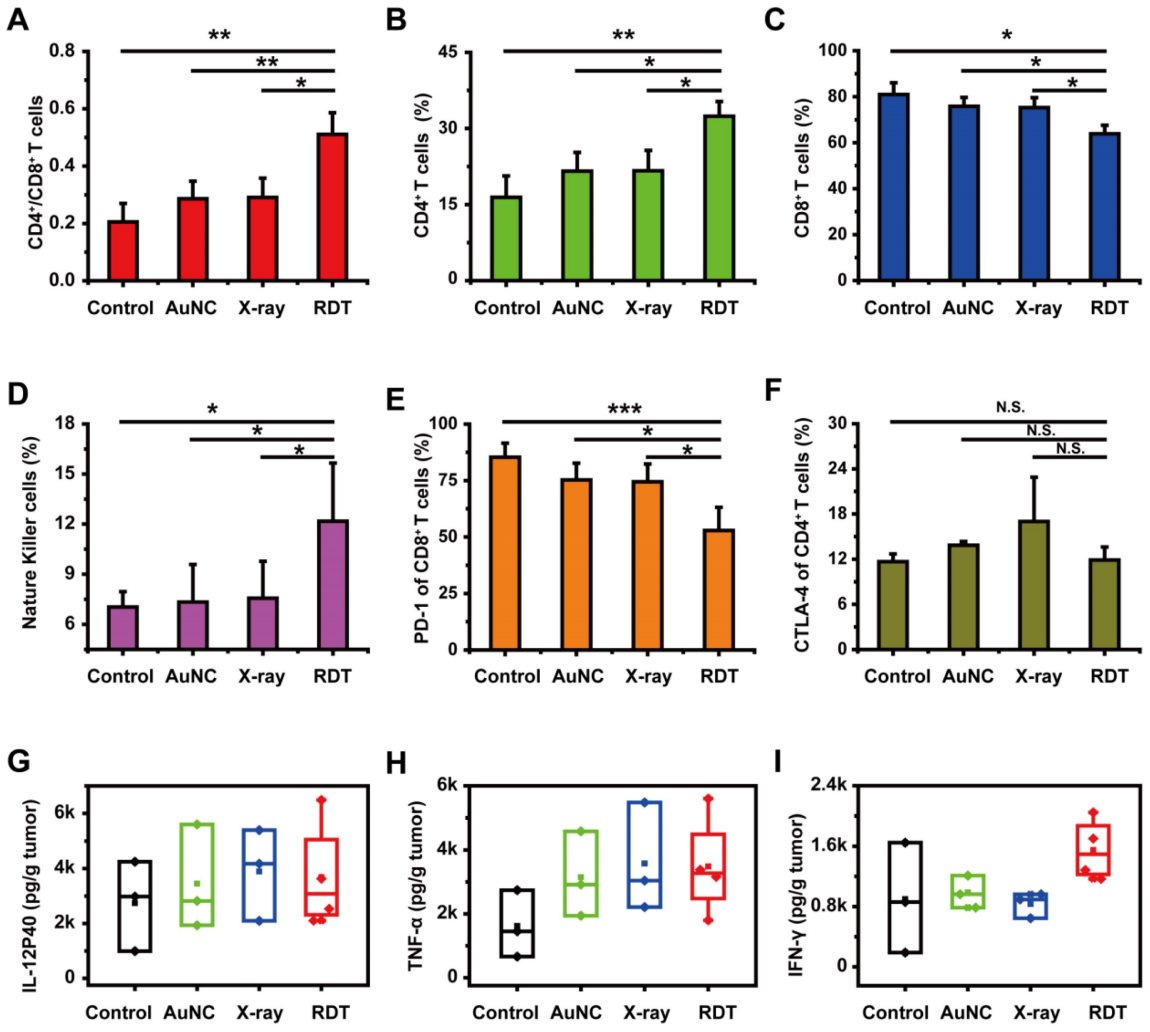
** Antitumor immune response of AuNC@DHLA RDT. (A)** Different groups of tumor-infiltrating CD4^+^/CD8^+^ T cells, **(B)** the percentage of tumor-infiltrating CD4^+^ T cells, **(C)** the percentage of tumor-infiltrating CD8^+^ T cells, **(D)** the percentage of tumor-infiltrating natural killer cells (NK), **(E)** the percentage of PD-1 expression on tumor-infiltrating CD8^+^ T cells and **(F)** CTLA-4 expression on tumor-infiltrating CD4^+^ T cells. Except that NK data analyzed by single-tailed t-test, all other data were analyzed by two-tailed t-test ((***p < 0.001, **p < 0.01, *p < 0.05, N.S. *p* > 0.05). **(G)** The secretion of interleukin-12p40 (IL-12P40), **(H)** tumor necrosis factor α (TNF-α) and** (I)** interferon γ (IFN-γ) in tumor sites in different groups. All data were analyzed by One-way ANOVA.

Several previous studies have shown that enhanced antitumor immunity, induced by immunogenic cell death and *in-situ* vaccination, could be observed during RDT 23, 54. For example, Lu *et al*. 55 combined the nanoscale metal-organic frameworks (NMOFs)-based RT-RDT therapy with checkpoint blockade immunotherapy and synergistic immunological responses had been achieved to eradicate distant tumors via consistent abscopal effect as well as inhibit tumor recurrence via antitumor immune memory. As for our system, the tumor-infiltrating CD4^+^/CD8^+^ ratio was determined by FACS assay and used for evaluating the immune response. As shown in Figure 6A, the CD4^+^/CD8^+^ ratio in the RDT group was significantly elevated to 0.51 ± 0.08 from 0.20 ± 0.06 in the control group, indicating the enhanced cell immune function. Similar phenomena were also observed in low-dose radiotherapy (LDRT) 56, 57, and thus LDRT was known as a potential immune amplifier capable of reprogramming the tumor microenvironment, instigating inflammation, and sensitizing 'cold' tumors to immune checkpoint blockade responsiveness 57.

Tumor-infiltrating immune cells, such as CD4^+^ T cells, CD8^+^ T cells, natural killer (NK) cells, B cells, macrophages, dendritic cells (DCs) and T regulatory (Treg) cells were detected for further evaluating the antitumor immune response. As shown in Figure 6B, 6D and Figure S11D, the percentages of CD4^+^ T cells, NK cells and DCs in RDT-treated tumor tissues were significantly higher than those from other groups. Specifically, after treatment with AuNC@DHLA RDT, the percentages of CD4^+^ T cells (Figure 6B), NK cells (Figure 6D) and DCs (Figure S11D) significantly increased to 32.40 ± 2.92%, 12.17 ± 3.48% and 5.00 ± 1.53% from 16.43 ± 4.20%, 7.03 ± 0.92% and 2.73 ± 1.42% in the control group, respectively. Interestingly, although the percentage CD8^+^ T cells in RDT group was slightly less than the other groups (Figure 6C), it did not affect the effects of AuNC@DHLA RDT. Although CD8^+^ T cells can directly suppress tumor growth, recent report has proved that CD4^+^ T cells not only block cancer cell cycle progression at G1/S, but also inhibited HER2^+^ breast cancer growth *in vivo* 58. As for T cells, B cells, macrophages and Treg cells, as shown in Figure S11A-C and Figure S11E, there were no apparent differences between the RDT group and other treated groups. These results indicated that AuNC@DHLA RDT has its strong ability of increasing activated immune effector cells and delivering these cells into tumor tissues, which is crucial for achieving tumor regression and abscopal effect 59.

Besides that, the percentages of PD-1 in the AuNC@DHLA RDT group significantly decreased to 52.87 ± 10.25% from 85.30 ± 6.25% in the control group (Figure 6E), while the percentage of CTLA-4 exhibited no obvious difference between the RDT group and other treated groups (Figure 6F). This decrease of PD-1 results in increased survival and proliferation of T cells, enhanced cytokines production and finally promotes an antitumor immune response 60. Cytokines, such as TNF-α and IL-12, are vital for the antitumor activity (Figure 6G-H). For example, interferon-γ (IFN-γ) has been proved to be essential for NK cells accumulation in tumors and stimulation of antitumor immune-response (Figure 6I) 61, 62. Although no significant differences (One-way ANOVA, *p* > 0.05) were observed among these groups, the cytokine content in the RDT group was higher than that in the control group. Specifically, after treatment with AuNC@DHLA RDT, the content of IL-12, TNF-α, and IFN-γ increased to 3688.14 ± 1973.12, 3484.09 ± 1572.89, and 1549.79 ± 401.87 from 2742.47 ± 1644.24, 1617.16 ± 1049.26, and 899.19 ± 730.63 in the control group, respectively. These increased cytokines production confirmed an enhanced antitumor immune response during AuNC@DHLA RDT process.

**Figure 7:**
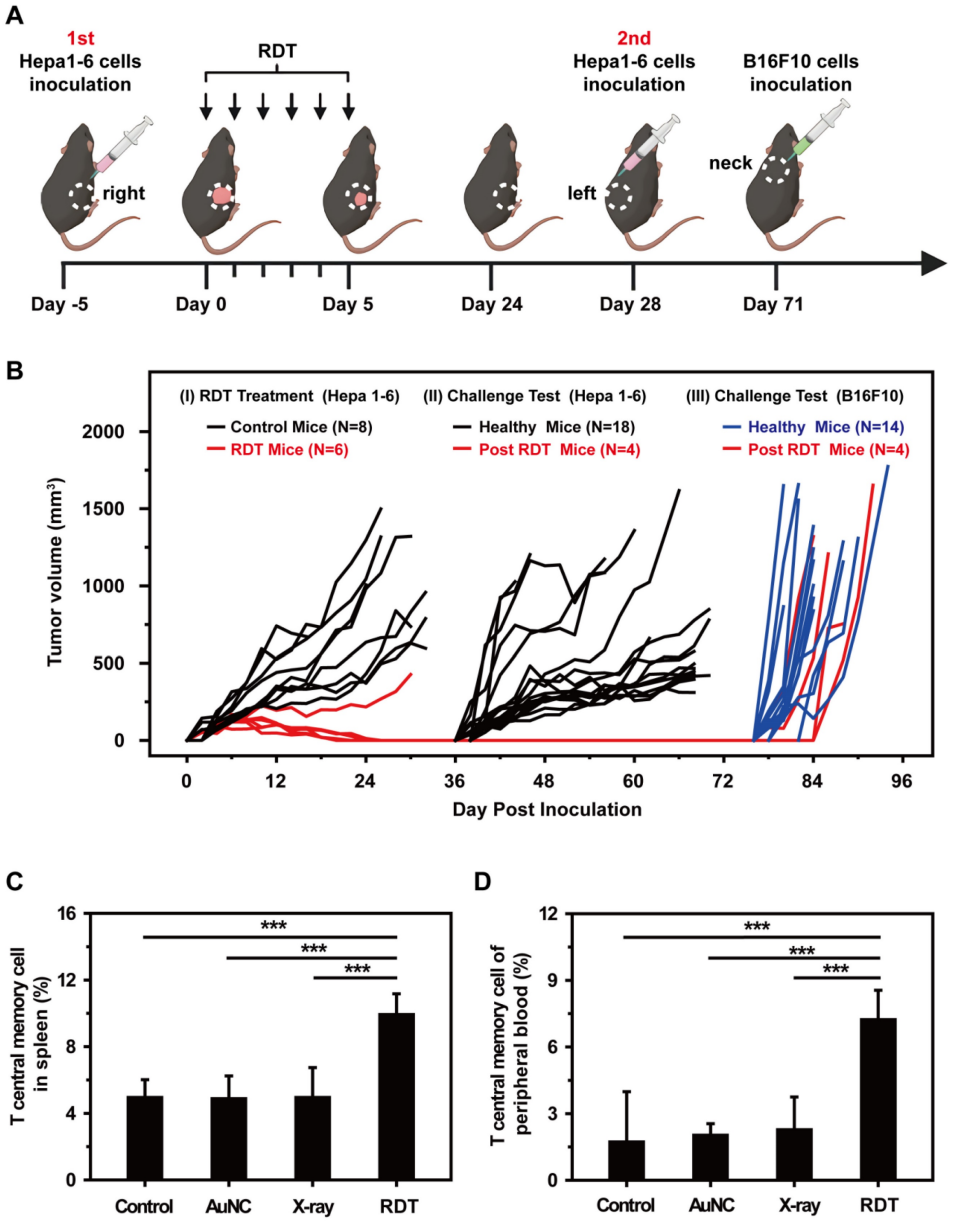
** (A)** Schematic illustration showing the design of animal experiments. **(B)** Tumor growth curves in the tumor challenge study. The tumor size of each mouse was plotted separately in control (black and blue curves) and RDT group (red curves), showing that five out of six mice in RDT group were tumor free after treatment. The black and blue curves showed the tumor growth curves of three control groups with Hepa 1-6 (black) or B16F10 (blue) tumor cells injected on the same days. **(C)** Spleen-infiltrating T central memory cells and **(D)** T central memory cells of peripheral blood in different groups on the 15th day after RDT treatment. Data were analyzed by two-tailed t-test (***p < 0.001).

Then, a tumor challenge test was performed in the survived mice of RDT group to determine whether anti-tumor immunity to the Hepa1-6 tumor was established. As shown in Figure 7A, the mice were firstly inoculated subcutaneously in the right flanks with Hepa1-6 cells and then treated with AuNC@DHLA RDT (Figure S12A). For five of the six mice, the tumors were completely eradicated on day 24 (Figure 7A-B). Then these four cured mice were then challenged with Hepa1-6 cells in the left flanks. In addition, other healthy mice (N = 18) were also inoculated as the control group. As shown in Figure 7B, all the 4 survived mice remained tumor free on both flanks until B16F10 cells challenge on day 71. In contrast, for all 18 healthy mice in the control group (Figure 7B) and mice in the X-ray group (Figure S12B), tumors grew normally. These results indicated a strong anticancer immune memory effect to Hepa 1-6 tumor was established during AuNC@DHLA RDT process. The 4 survived mice were further challenged with B16F10 cells to estimate the immune specificity. On day 71, B16F10 cells (5 × 10^6^ per mouse) were inoculated on the back of neck of the 4 survived mice, with 14 healthy as control mice. As shown in Figure 7B, tumors grew normally on both the 4 treated mice and 14 healthy control mice. And the tumor growth curves showed no differences between these groups. These results indicate that the antitumor immune memory effect induced during AuNC@DHLA RDT process is highly specific. This immune memory effect may be ascribed to the enhanced T central memory cell, which home to the T cell region of secondary lymphoid organs, remain in the body for a long time, have little or no effector function, but readily proliferate and differentiate into effector cells in response to antigenic stimulation. As shown in Figure 7C-D, the percentages of T central memory cells (Tcm) in spleen and in peripheral blood from the RDT group were significantly increased to 9.93 ± 1.24% and 7.22 ± 1.33% from 4.95 ± 1.07% and 1.73 ± 2.26% in the control group on the 15th day after RDT treatment. While there was no obvious difference in the percentage of spleen-infiltrating T effector memory cells (Tem) between RDT group and other control groups (Figure S13A), but as for in peripheral blood sample, the percentage of Tem in RDT group was significantly lower than other control groups (Figure S13B). Furthermore, AuNC@DHLA could activate immature dendritic cells deriving from bone marrow (BMDCs) of C57BL/6j mice (Figure S14A-D). Previous studies proved that nanoparticles favor the maturation process of DCs, and smaller nanoparticles have stronger capability than bigger ones 63-65. Take into account the ultra-small size of AuNC@DHLA, enhanced antitumor immune response and increased efficacy can be expected.

### *In vivo* toxicity

**Figure 8:**
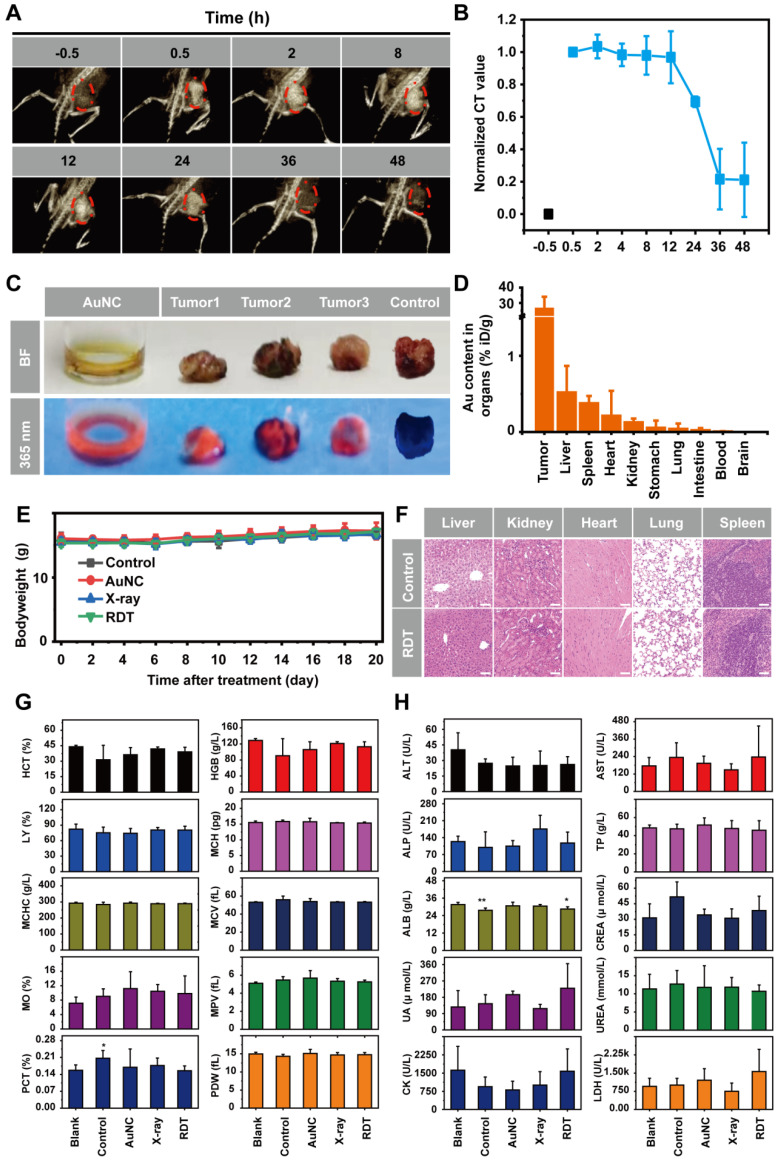
***In vivo* toxicity studies of AuNC@DHLA RDT. (A)*** In vivo* 3D CT images of tumor-bearing mice before and at 0.5, 2, 8, 12, 24, 36, and 48 h after intratumoral injection of AuNC@DHLA (10 mg mL^-1^, 100 μL). The tumor is indicated by red dotted lines. **(B)** Statistics of *in vivo* pharmacokinetic process of AuNC@DHLA. **(C)** AuNC@DHLA aquatos solution and tumor treated with and without AuNC@DHLA under natural light (BF: bright filed) and under 365 nm at 48 h post injection. **(D)** Biodistribution of AuNC@DHLA in the main organs at 48 h post injection, as determined by ICP-MS. Data are presented as mean ± SD. N = 3. **(E)** Body weight curves of mice that received different treatments. No obvious loss of body weight was observed in all the groups.** (F)** Slices of H&E staining containing liver, kidney, heart, lung, and spleen of tumor-bearing mice on the 20th day after treatment with PBS and RDT. Scale bar: 50 µm. **(G)** Hematology data of mice with different treatments on day 20. The results show mean and standard deviation of hematocrit (HCT%), hemoglobin (HGB), lymphocyte ratio (LY%), mean corpuscular hemoglobin (MCH), mean corpuscular hemoglobin concentration (MCHC), mean corpuscular volume (MCV), monocyte rate (MO%), mean platelet volume (MPV), plateletcrit (PCT%), platelet distribution width (PDW). Data were analyzed by two-tailed t-test (*p < 0.05). **(H)** Blood biochemistry analysis of mice with different treatments on day 20. The results show mean and standard deviation of alanine aminotransferase (ALT), aspartate aminotransferase (AST), alkaline phosphatase (ALP), total protein (TP), albumin (ALB), creatinine (CREA), uric acid (UA), urea (UREA), creatine kinase (CK), and lactic dehydrogenase (LDH). Data were analyzed by two-tailed t-test (**p < 0.01, *p < 0.05).

The potential *in vivo* toxicity is also an important issue in the application of nanomaterial therapeutic system. Ideal nanomaterials should be effectively removed from the body (renal clearable), with little nonspecific accumulation in organs and minimal potential health hazards. The removal efficiency of nanomaterial therapeutic system from the body is primarily dependent upon particle size, charge, surface modification, and coated ligand 26, 27, 66, 67. Prior to *in vivo* AuNC@DHLA RDT efficacy, pharmacokinetic studies of AuNC@DHLA were firstly conducted by X-ray computed tomography (CT) imaging. After testing the stability of CT imaging system by AuNC@DHLA in pork tissue (Figure S15A-D), the mice were intratumorally injected with AuNC@DHLA at the concentration of 10.0 mg mL^-1^, and then was monitored for 48 h (Figure 8A). As shown in Figure 8B, the concentrations of AuNC@DHLA in the region of tumor, sharply increased during the first 0.5 to 2.0 h after dosing, slightly decreased or plateaued between 2.0 and 12 h, and subsequently decreased. As for the region of tumor, bright red fluorescence could be obviously observed at 48 h after intratumoral injection with AuNC@DHLA (Figure 8C). Meanwhile, AuNC@DHLA preferentially accumulated in the liver, as the green arrow indicated, during the first 24 h post injection and gradually disappeared in the following time (Figure S16). Further quantitative analysis with inductively coupled plasma mass spectrometry (ICP-MS) indicated that more AuNC@DHLA stayed at the tumor region than other organs, such as liver and spleen, as shown in Figure 8D. And no AuNC@DHLA was observed in the brain at 48 h after administration. These above results demonstrated that AuNC@DHLA mainly accumulated in the region of tumor, and rarely accumulated in other organs. Presumably, since the AuNC@DHLA are smaller than the glomerular filtration cutoff (~ 6 nm) 26, they escape capture by the reticuloendothelial system (RES) and are excreted via the urinary system, limiting potential toxicity to the RES organs. The decreased pH (acidosis) within TME may account for this tumor accumulation, due to the decreased electrostatic repulsion and increased hydrogen bonding, as well as attractive van der Waals forces 67.

Individual body weights were recorded at different time points post treatment (Figure 8E). For all these treatment groups and the control group, no significant body weight changes were observed during the whole treatment period (Figure 8E), indicating that no significant systemic toxicity appeared. Moreover, further histological examinations of liver, kidney, heart, lung, and spleen also indicated no apparent abnormalities or lesions after AuNC@DHLA RDT treatment (Figure 8F). Hematology and blood biochemistry experiments were conducted on the 20th day post injection. No abnormal indicators of hematology were found in any of the treatment groups (Figure 8G). The hepatic-related serum chemistry analysis including alanine aminotransferase (ALT), aspartate aminotransferase (AST), alkaline phosphatase (ALP), total protein (TP), albumin (ALB), which was highly related to the liver damage and liver function alternation, showed no obvious change compared to healthy mice without tumor (Figure 8H). Blood biochemical tests also showed that the kidney and heart were functioning normally. These above results indicate that AuNC@DHLA RDT is a safe and highly effective system for *in vivo* cancer therapy.

## Conclusions

We constructed a safe and efficient RDT solely based on a nanomaterial, AuNC@DHLA, which could be directly excited by low-dose X-ray and further exhibited strong ability of ROS (O_2_^-•^ and HO•) generation. Moreover, even under hypoxic conditions, AuNC@DHLA demonstrated excellent radiodynamic performance. Highly efficient* in vivo* treatment of solid tumors had been achieved and enhanced antitumor immune response was also observed, which could be vital against tumor recurrence or metastasis. Negligible systemic toxicity was observed as a consequence of the ultra-small size of AuNC@DHLA and rapid body clearance. Taken together, our developed AuNC@DHLA RDT system paves the way for treatment of solid tumors, combining the advantages of superior tissue penetration depth, being directly excited by low-dose X-ray radiation, excellent radiodynamic performance even under hypoxic conditions, and negligible systemic toxicity. Although this work demonstrated herein is a proof-of-principle basic research on evaluation of AuNC@DHLA as an efficient RDT, use of an intravenous and lymphatic delivery system will be explored in an orthotopic model to further characterize the clinical potential of AuNC@DHLA through examination of pharmacokinetics and biodistribution, etc. We believe that our developed system will have important clinical application value as an effective therapeutic approach in treating patients with solid tumors, including hepatoma, breast cancer, and lung cancer, as well as adjuvant treatment, such as chemotherapy and immunotherapy.

## Supplementary Material

Supplementary methods, figures and tables.

## Abbreviations

RDT: radiodynamic therapy
PDT: photodynamic therapy
PS: photosensitizer
ROS: reactive oxygen species
AuNCs: gold nanoclusters
AuNC@DHLA: dihydrolipoic acid coated gold nanoclusters
AuNC@GSH: glutathione-protected gold nanolcusters
AO: acridine orange
RB: rose bengal
