# Supplementary material for: Low-dose X-ray radiodynamic therapy solely based on gold nanoclusters for efficient treatment of deep hypoxic solid tumors combined with enhanced antitumor immune response
Source: Theranostics. 2023 Jan 22;13(3):1042–58. doi: 10.7150/thno.78649 (PMC9925321; doi:10.7150/thno.78649)
Supplement: Supplementary file 1 — Supplementary methods, figures and tables. [file thnov13p1042s1.pdf]

Supplementary information

Low-dose X-ray radiodynamic therapy solely based on gold nanoclusters for efficient treatment of deep hypoxic solid tumors combined with enhanced antitumor immune response

Shengcang Zhu[1,2,#], Feihong Yan[1,2,#], Lulu Yang[3], Bingyi Li[1], Ruxian Xue[1,2], Wenwen Yu[1,2], Yu Wang[1,2], Lu Huang[1,2], Lijun Wang[3], Rongcheng Han[1,2,✉], and Yuqiang Jiang[1,2,✉]

1. State Key Laboratory of Molecular Developmental Biology, Institute of Genetics and Developmental Biology, Chinese Academy of Sciences, Beijing 100101, China

2. University of Chinese Academy of Sciences, Beijing 100049, China

3. Single-Molecule and Nanobiology Laboratory, Department of Biochemistry and Biophysics, School of Basic Medical Sciences, Peking University, Beijing 100191, China

[#] These authors contributed equally.

✉Corresponding authors: State Key Laboratory of Molecular Developmental Biology, Institute of Genetics and Developmental Biology, University of Chinese Academy of Sciences, Beijing 100049, China. Tel.: 010-64806362; E-mail: Dr. Rongcheng Han: hanrch@genetics.ac.cn, Dr. Yuqiang Jiang: yqjiang@genetics.ac.cn.

Characterization of AuNC@DHLA

The sizes and morphologies of the AuNC@DHLA were characterized by high-resolution TEM on a JEM-2100F (JEOL, Japan) with an acceleration voltage of 200 kV. UV−vis spectra were measured with a spectrophotometer (TU-1901, Puxi versatile instrument Co. China). Photo-luminescence spectra and excitation spectrum of AuNC@DHLA were recorded on a Hitachi FL-4500 spectrofluorometer. The hydrodynamic diameter (HD) distributions of the as-synthesized AuNC@DHLA were determined by dynamic light scattering (DLS) on a ZetaPALS instrument (Brookhaven). The DLS data were acquired in the phase analysis light scattering mode at 25 °C, and the sample solutions were prepared by dissolving the AuNC@DHLA in distilled water.

Cytotoxicity of AuNC@DHLA

For Hepa 1-6 cells, cytotoxicity measurement was performed using the IncuCyte S3 system. Hepa 1-6 cells were trypsinized and resuspended in DMEM containing 10% FBS and 1% penicillin-streptomycin. The cells were seeded at a density of 4000 cells per well in a 96-well plate. After 24 h of incubation at 37 °C in 5% CO_2, the cells were washed three times with PBS solution (pH 7.4). Colloidal solutions of AuNC@DHLA with different concentrations (100 μL) were added to the wells. Then cell growth was collected every six hours by IncuCyte S3 system.

For Huh-7 and 293T cells, cytotoxicity measurements were performed using the MTT (3-(4,5-dimethylthiazol-2-yl)-2,5-diphenyltetrazolium bromide) assay. Briefly, Huh-7 and 293T cells were seeded at a density of 4000 cells per well in a 96-well plate.

After 24 h of incubation at 37 °C in 5% CO_2, the cells were washed three times with PBS solution (pH 7.4). Colloidal solutions of AuNC@DHLA with different concentrations (100 μL) were added to the wells. After 24 h of incubation at 37 °C in 5% CO_2, a solution of MTT (50 μL, 5.0 mg mL^{-1}) was added to each well. After 4 h of incubation at 37 °C in 5% CO_2, the medium was discarded and the intracellular precipitate of formazan was collected by the addition of DMSO (100 μL). The absorbance at 570 nm was measured on a Bio-Rad model 550 microplate reader. Each data point was collected by averaging six wells, and the untreated cells were used as controls.

Cell cycle analysis

A BeyoClick™ EDU-488 Cell Proliferation Detection Kit (Beyotime, China) and DAPI were used to measure the cell cycle distribution. After incubating with AuNC@DHLA at 200 μg mL^{-1} for 2 h, the cells without and with irradiation (1.0 Gy) were incubated at 37 °C for 2 days. After treating with BeyoClick™ EDU-488 Cell Proliferation Detection Kit, the cells were stained with DAPI for 10 min under darkness. The cell cycle distribution was analyzed by flow cytometry.

Tumor-infiltrating immune cells

Tumor tissues were harvested from mice in different groups, cut up and next removed red cells by red blood cell lysis buffer. Those remaining cells were labeled by anti-FOXP3-Alexa Fluor®647 (Biolegend, Clone: MF-14, Catalog: 126408), anti-MHC II-APC (Dogesce, Clone: M5/114.15.2, Catalog: 17-5321-81), anti-CD11c-

eFluor 450 (eBioscience, Clone: N418, Catalog: 48-0114-82), anti-F4/80-Brilliant Violet 711™ (eBioscience, Clone: BM8, Catalog: 123147), anti-CD45R (B220)-PE-Cyanine7 (eBioscience, Clone: RA3-6B2, Catalog: 25-0452-82), anti-NK1.1-PE (eBioscience, Clone: PK136, Catalog: 12-5941-82), anti-CD4-eFluor 506 (Thermofisher, Clone: RM4-5, Catalog: 69-0042-82), anti-CD8a-APC-Cyanine7 (Biolegend, Clone: 53-6.7, Catalog: 100714), anti-CD3-PerCP-eFluor 710 (eBioscience, Clone: 17A2, Catalog: 46-0032-82), anti-CD45-FITC (eBioscience, Clone: 30-F11, Catalog: 11-0451-82) antibodies according to the manufacturer's protocols. All these antibodies used in our experiments were diluted for 100 times. T cells were labelled by $CD45^+$ $CD3^+$. B cells were labelled by $CD45^+$ $CD3^-$ $B220^+$. Macrophages were labelled by $CD45^+$ $CD3^-$ $B220^-$ $F4/80^+$. DCs were labelled by $CD45^+$ $CD3^-$ $B220^-$ $F4/80^-$ $CD11c^+$ MHC II^+. Treg cells were labelled by $CD45^+$ $CD3^+$ $CD4^+$ $FOXP3^+$.

Marrow-derived dendritic cells (BMDCs)

Bone marrow-derived dendritic cells (BMDCs) with and without AuNC@DHLA treatment were labeled by anti-CD86-FITC (eBioscience, Catalog: 11-0862-82), anti-CD80-PE (eBioscience, Catalog: 12-0801-82), and anti-MHC II-APC (Dogesce, Clone: M5/114.15.2, Catalog: 17-5321-81) antibodies according to the manufacturer's protocols. All these antibodies used in our experiments were diluted for 100 times. Mature BMDCs were labelled by MHC II^+ $CD86^+/CD80^+$.

T effector memory cells

Spleen tissues were harvested from mice in different groups, then were crushed, and next removed red cells by red blood cell lysis buffer. Those remaining cells were labeled by anti-CD44-APC (eBioscience, Clone: IM7, Catalog: 17-0441-82), anti-CCR7-PE (abcam, Clone: 4B12, Catalog: ab95669), anti-CD62L-APC (abcam, Clone: MEL-14, Catalog: ab41459), anti-CD8a-APC-Cyanine7 (Biolegend, Clone: 53-6.7, Catalog: 100714), anti-CD3-PerCP-eFluor 710 (eBioscience, Clone: 17A2, Catalog: 46-0032-82), and anti-CD45-FITC (eBioscience, Clone: 30-F11, Catalog: 11-0451-82) antibodies according to the manufacturer's protocols. All these antibodies used in our experiments were diluted for 100 times. T effector memory cells were labelled by $CD45^{+}$ $CD3^{+}$ $CD8^{+}$ $CD44^{+}$ $CCR7^{-}$ $CD62L^{-}$.

T effector memory cells in peripheral blood were detected using the same method described above, excepting for the step of being crushed.

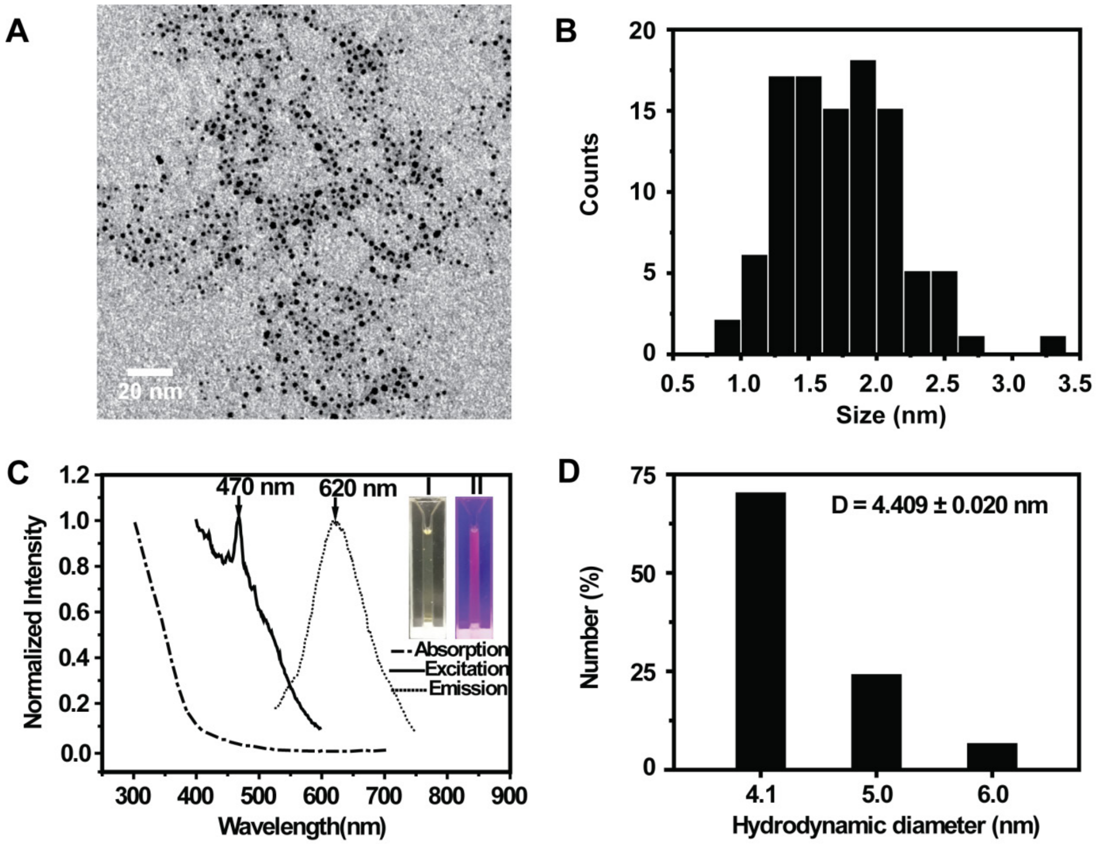

Figure S1. (A) Transmission electron microscopy (TEM) of AuNC@DHLA and (B) corresponding particle size distribution. (C) Absorption spectrum, excitation spectrum and photoluminescence spectrum of AuNC@DHLA. Insert: the picture of AuNC@DHLA aqueous solution under natural light (I) and 365 nm UV light (II). (D) The hydrodynamic diameter of AuNC@DHLA determined by dynamic light scattering (DLS).

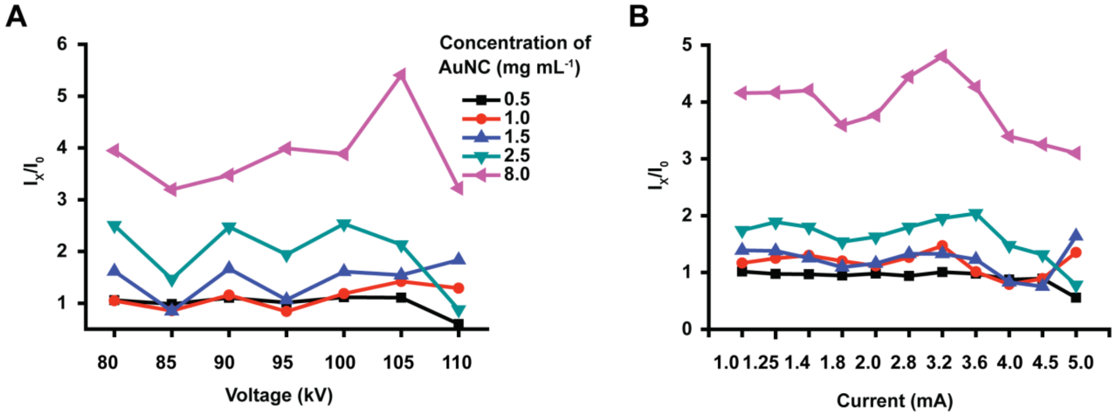

Figure S2. (A) Different tube voltages (under 5.0 mA current) and (B) Different tube currents (under 110 kV voltage) were used to explore the optimal conditions for imaging of AuNC@DHLA aqueous solution at different concentrations with 5 mm thick aluminum plate barrier. The legend of Figure S2B is the same to that of Figure S2A. I_0: X-ray absorption value of gold nanoclusters at a concentration of 0.5 mg mL^{-1}; I_X: X-ray absorption values of gold nanoclusters with different concentrations from 0.5 to 8.0 mg mL^{-1}.

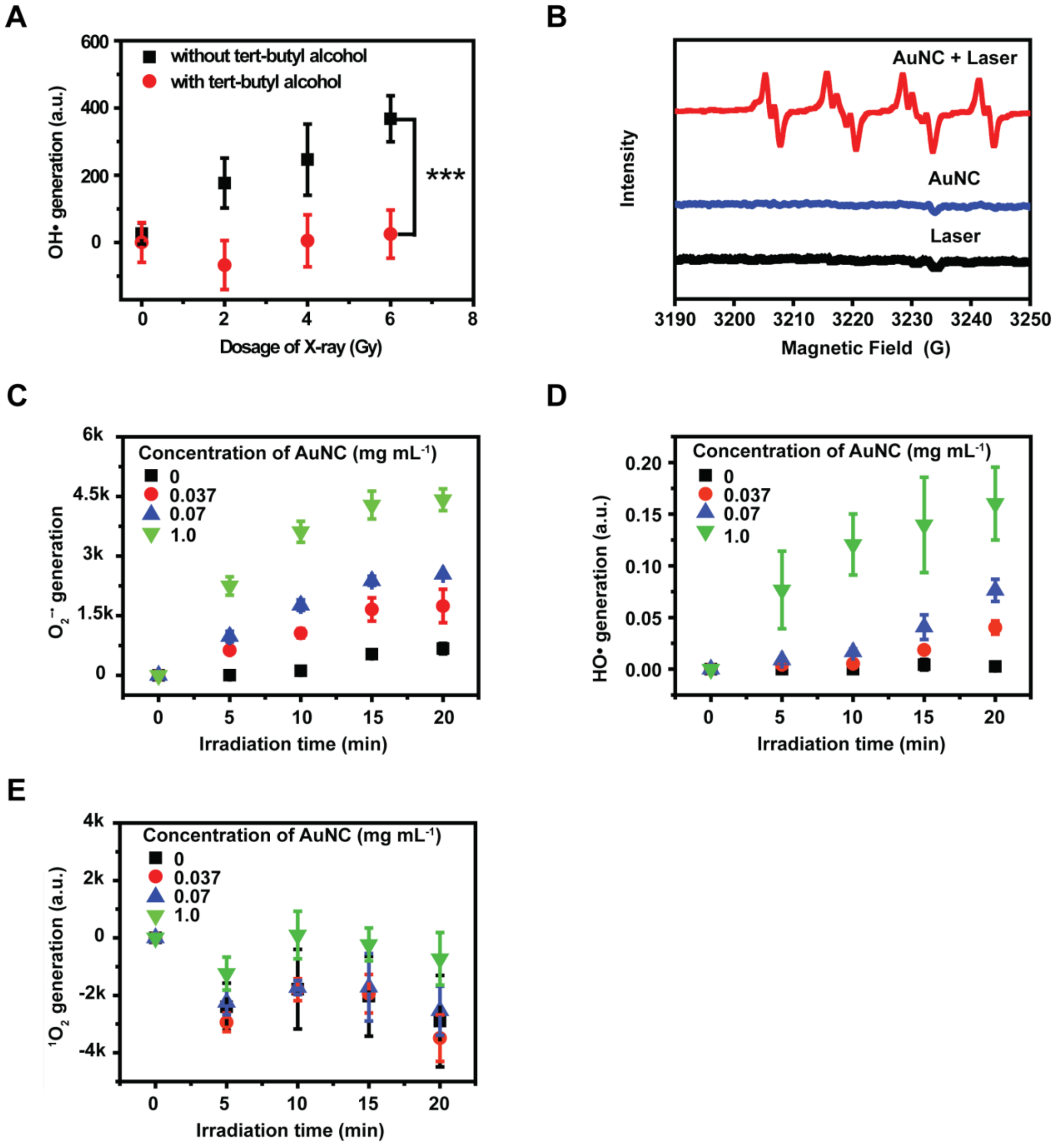

Figure S3. (A) HO• generation without and with tert-butyl alcohol under different dosage of X-ray. The concentration of AuNC was 500 μg mL^{-1}. Statistical analysis was

performed by two-tailed t-test (***$p < 0.001$). (B) ESR spectra of superoxide radical ($O_2^{-\bullet}$) spin adduct (DMPO-OOH) in DMSO containing 0.1 M DMPO, generated by AuNC@DHLA (1.0 mg mL^{-1}) under a blue (400−1100 nm) Xenon light (20 mA) for 15 s. (C) $O_2^{-\bullet}$ generation, (D) HO• generation, and (E) $^1O_2$ generation of AuNC@DHLA under two-photon excitation with 800 nm fs laser (60.8 W cm^{-2}).

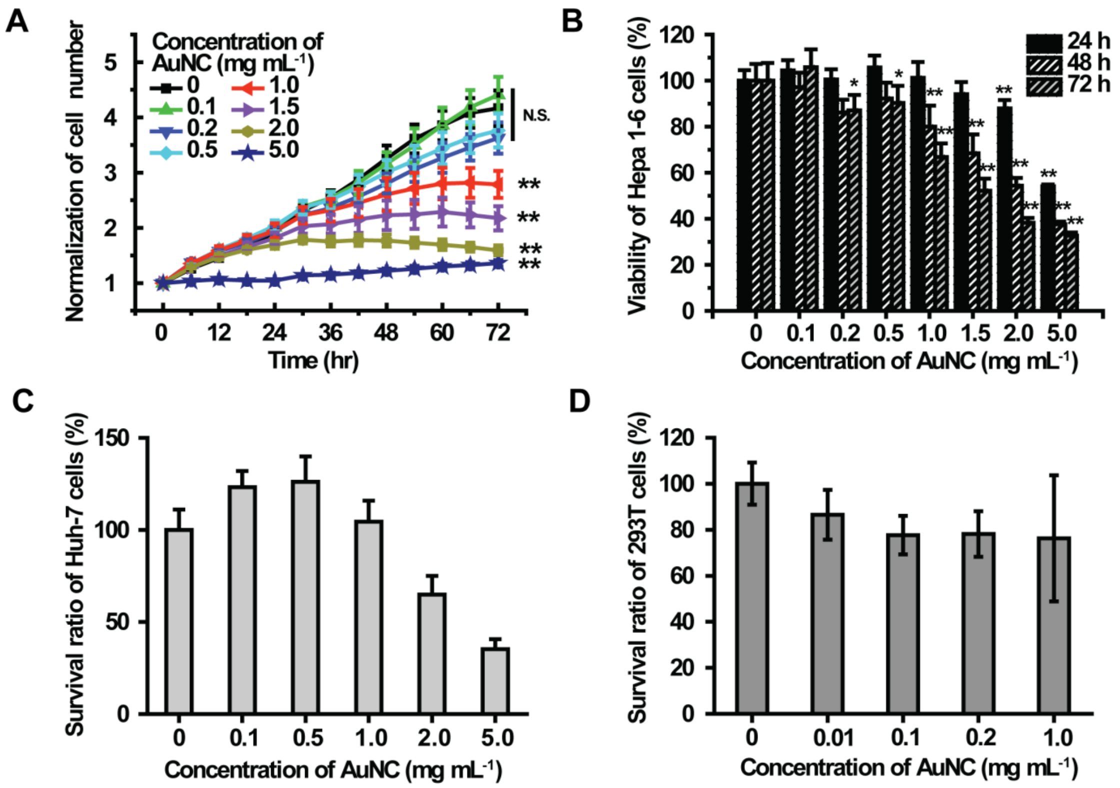

Figure S4. (A) The proliferation and (B) the survival rate of Hepa 1-6 cells at different concentrations of AuNC@DHLA in dark condition. The survival rate of (C) Huh-7 cells and (D) 293T cells by MTT method.

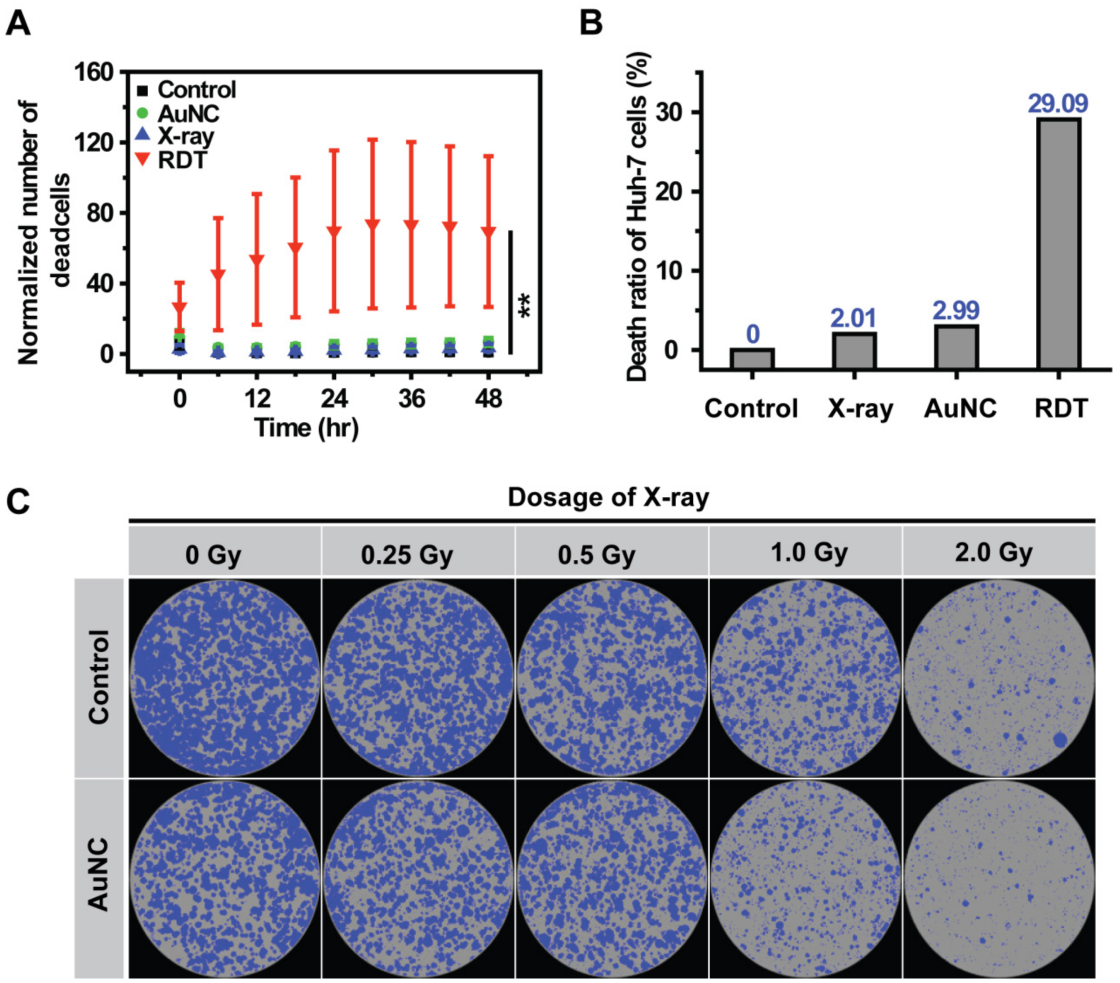

Figure S5. (A) Dead number of Hepa 1-6 cells and (B) death ratio of Huh-7 cells as measured by propidium iodide (PI) after receiving different treatments (control group, AuNC group, X-ray group (1.0 Gy), RDT group. The concentration of AuNC@DHLA was 200 μg mL^{-1}. Statistical analysis was performed by two-tailed t-test (***p* < 0.01). (C) Representative images of the colony formation assay of 293T cells with different treatments under normoxic condition (21% O_2).

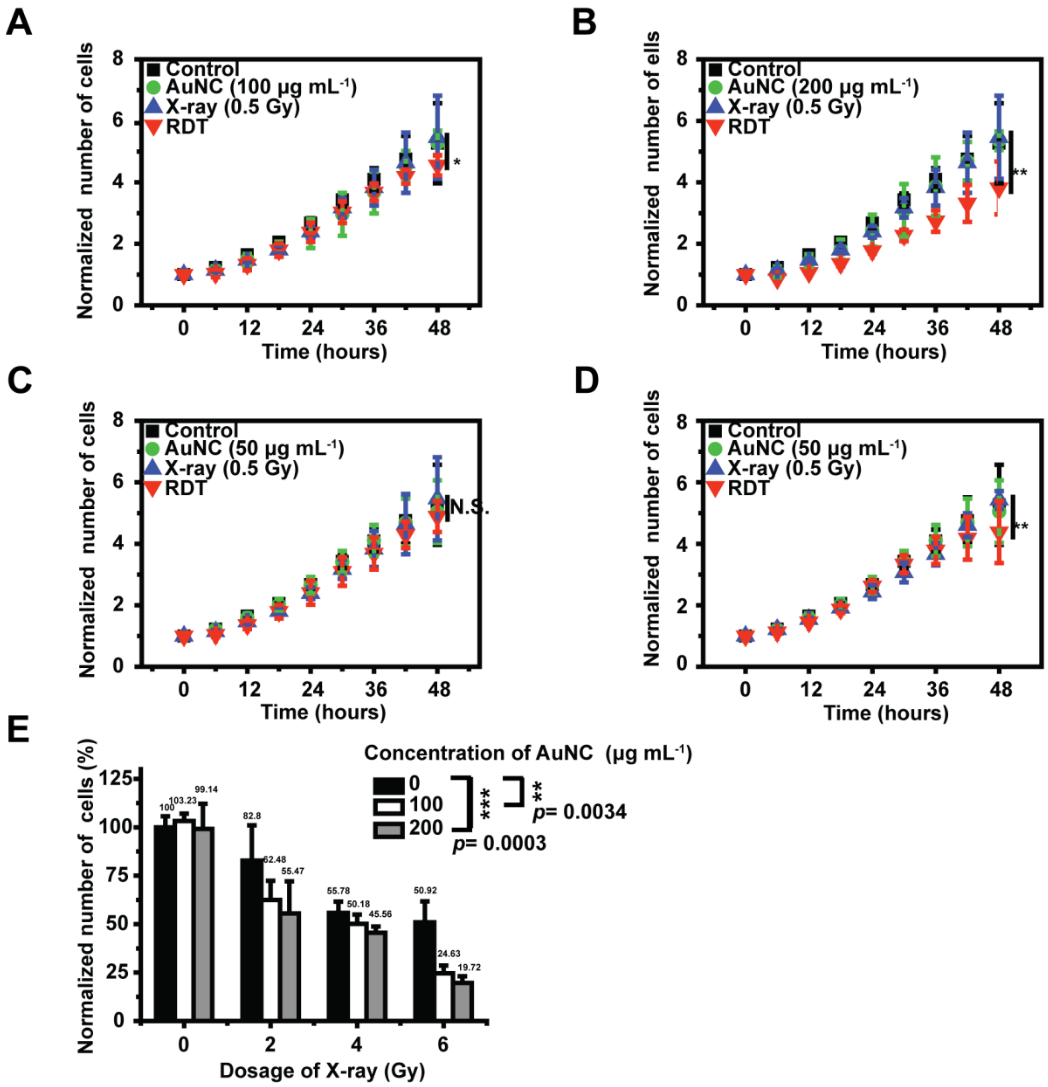

Figure S6. AuNC@DHLA RDT could efficiently inhibit cell division of Hepa 1-6 cells. (A) The inhibition of cell division induced by AuNC@DHLA (100 μg mL^{-1}) and X-ray (0.5 Gy) with a dose rate of 1.0 Gy min^{-1}. (B) The inhibition of cell division induced by AuNC@DHLA (200 μg mL^{-1}) and X-ray (0.5 Gy) with a dose rate of 1.0 Gy min^{-1}. (C) The inhibition of cell division induced by AuNC@DHLA (50 μg mL^{-1}) and X-ray (0.5 Gy) with a dose rate of 1.0 Gy min^{-1}. (D) The inhibition of cell division induced by AuNC@DHLA (50 μg mL^{-1}) and X-ray (0.5 Gy) with a dose rate of 3.8 Gy min^{-1}. For comparison, the blank control, the AuNC@DHLA alone without X-ray and X-ray alone without AuNC@DHLA, were also shown. (E) Statistical result of Hepa 1-6 cells after receiving different treatments. Statistical analysis was performed by two-tailed t-test (**$p < 0.01$, ***$p < 0.001$).

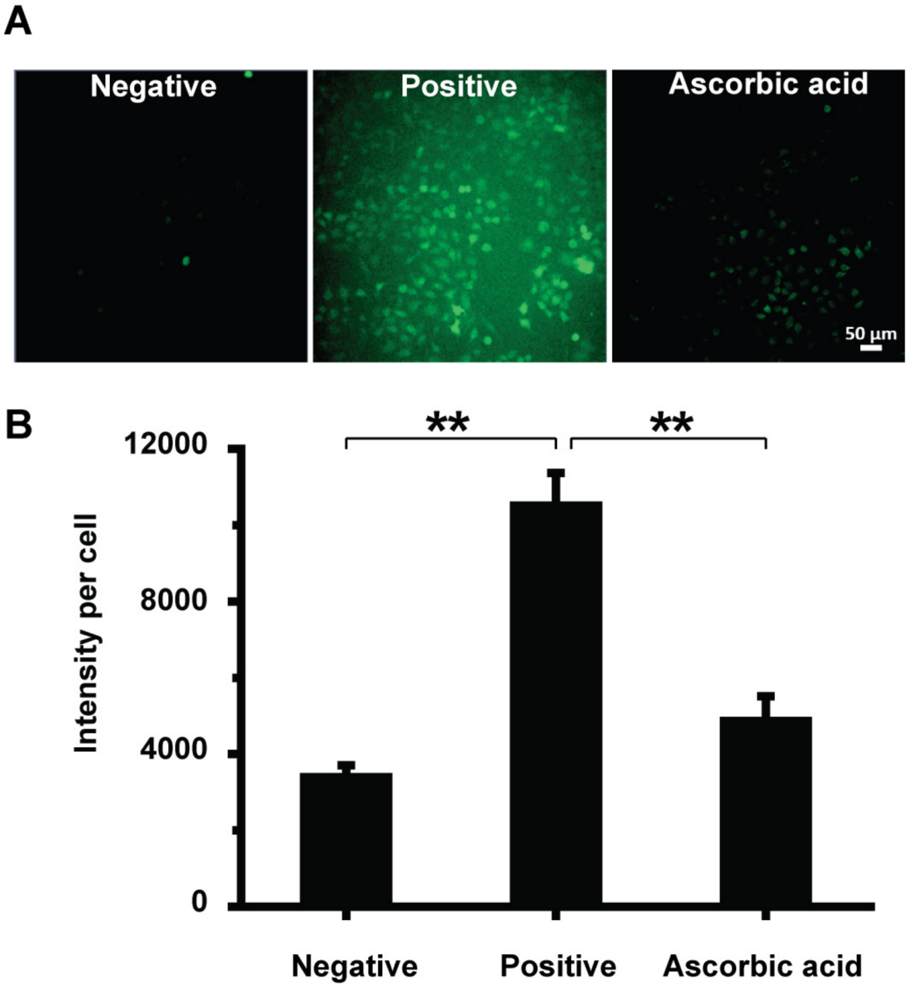

Figure S7. (A) Fluorescence intensity of Hepa 1-6 cells (Negative), treated with H_2O_2 (Positive) and treated with H_2O_2 and ascorbic acid (vitamin C). (B) Statistics of fluorescence intensity of different group. Data were analyzed by two-tailed t-test (**$p < 0.01$).

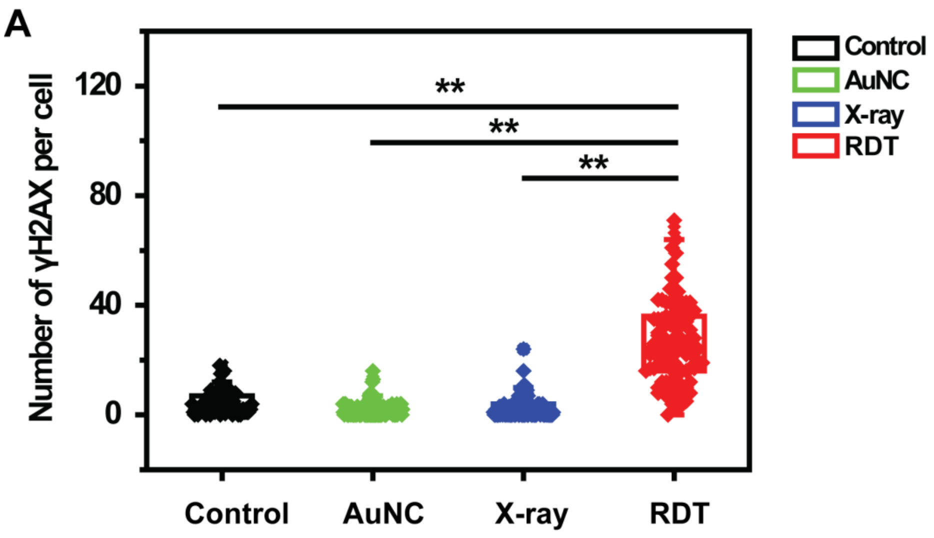

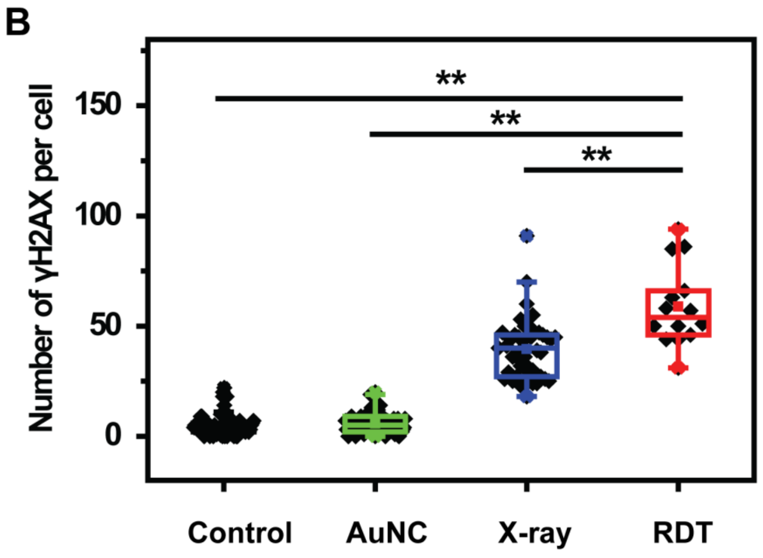

Figure S8. DNA damage of AuNC@DHLA RDT with (A) 0.5 Gy and (B) 6 Gy doses of X-ray in Hepa 1-6 cells. N ≥ 12. The concentration of AuNC@DHLA was 200 μg mL^{-1}. Statistical analysis was performed by two-tailed t-test (**$p < 0.01$).

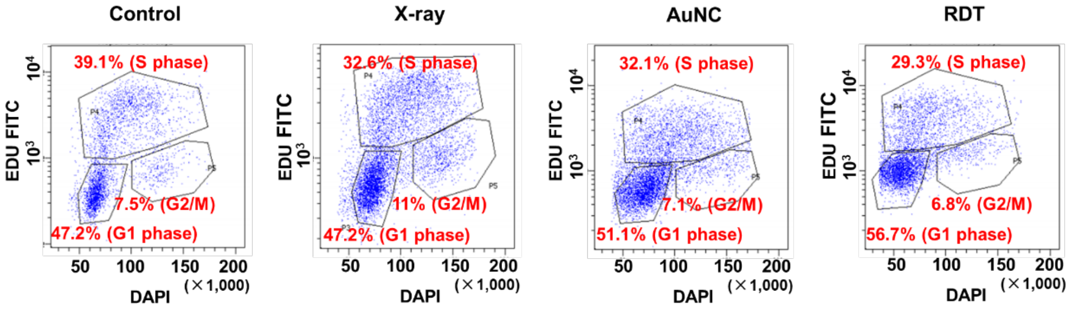

Figure S9. Cell cycle of Hepa 1-6 distribution of the control group, X-ray group, AuNC group and RDT group. The concentration of AuNC@DHLA was 200 μg mL^{-1}. Note that the percentage of G1 phase cell increased from 47.2% in the control group to 56.7% in the RDT group. Meanwhile, the percentage of S phase cell decreased from 39.1% in the control group to 29.3% in the RDT group. These results indicate that cells were arrested on the G1/S checkpoint [2].

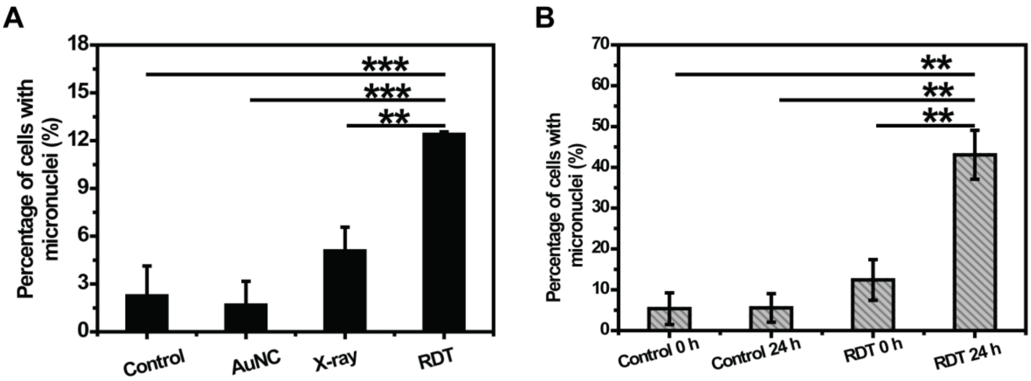

Figure S10. (A) The percentage of Hepa 1-6 cells with micronuclei in AuNC@DHLA RDT with 6.0 Gy doses of X-ray irradiation within 1 h and (B) control and RDT groups after 0 and 24 h. The concentration of AuNC@DHLA was 200 μg mL^{-1}. Data were analyzed by two-tailed t-test (***$p < 0.001$,**$p < 0.01$).

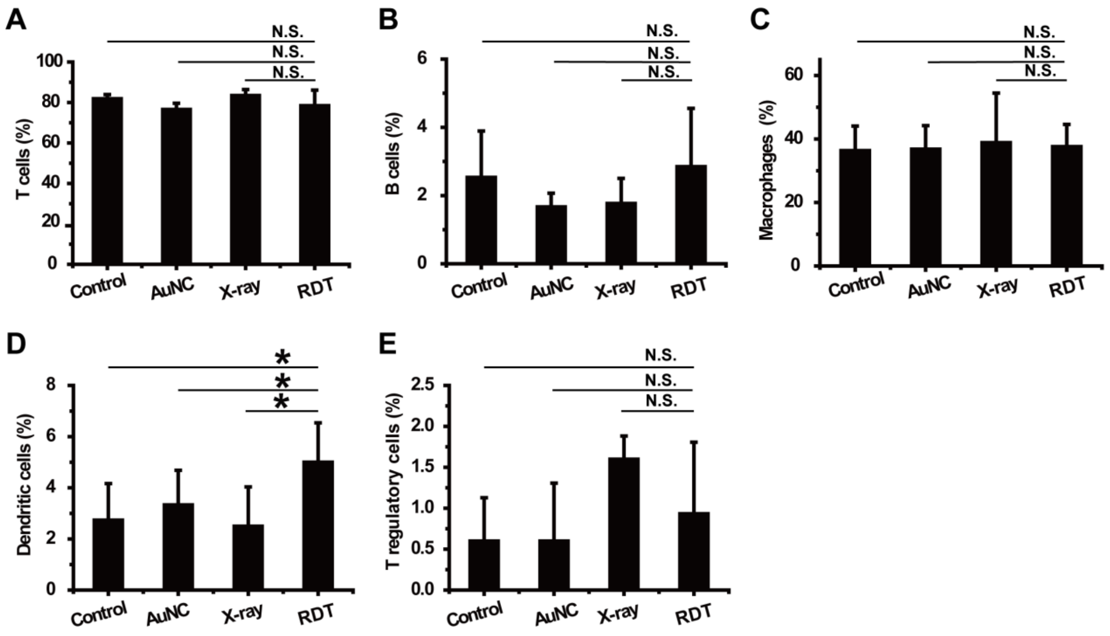

Figure S11. (A) The percentage of tumor-infiltrating T cells, (B) the percentage of tumor-infiltrating B cells, (C) the percentage of tumor-infiltrating macrophages, (D) the percentage of tumor-infiltrating dendritic cells and (E) the percentage of tumor-infiltrating T regulatory cells. Data were expressed as mean ± SD. Data were analyzed by two-tailed t-test (*$p < 0.05$, N.S. $p > 0.05$).

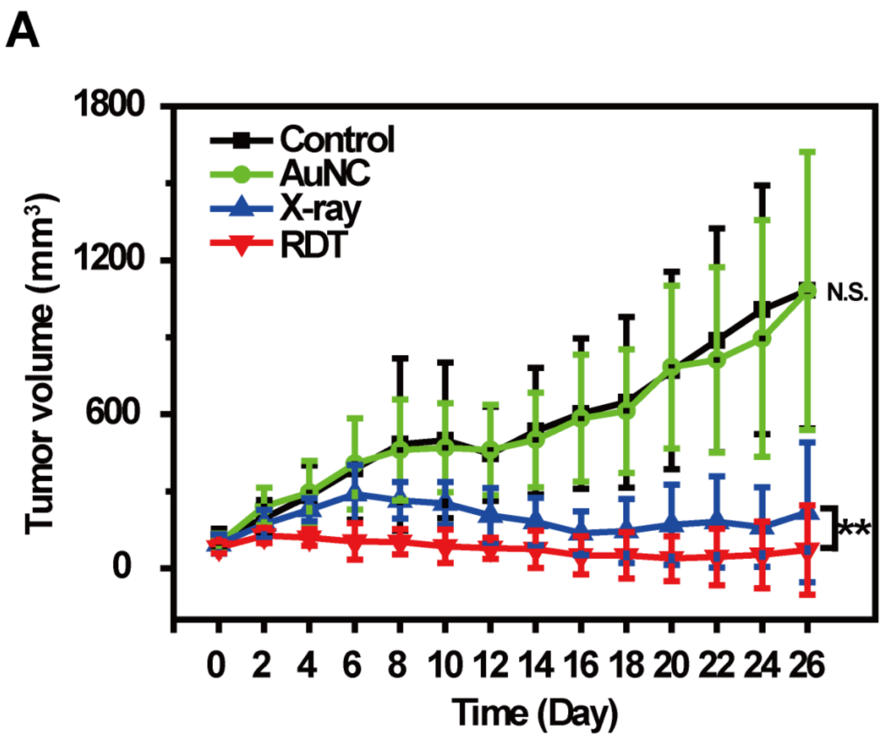

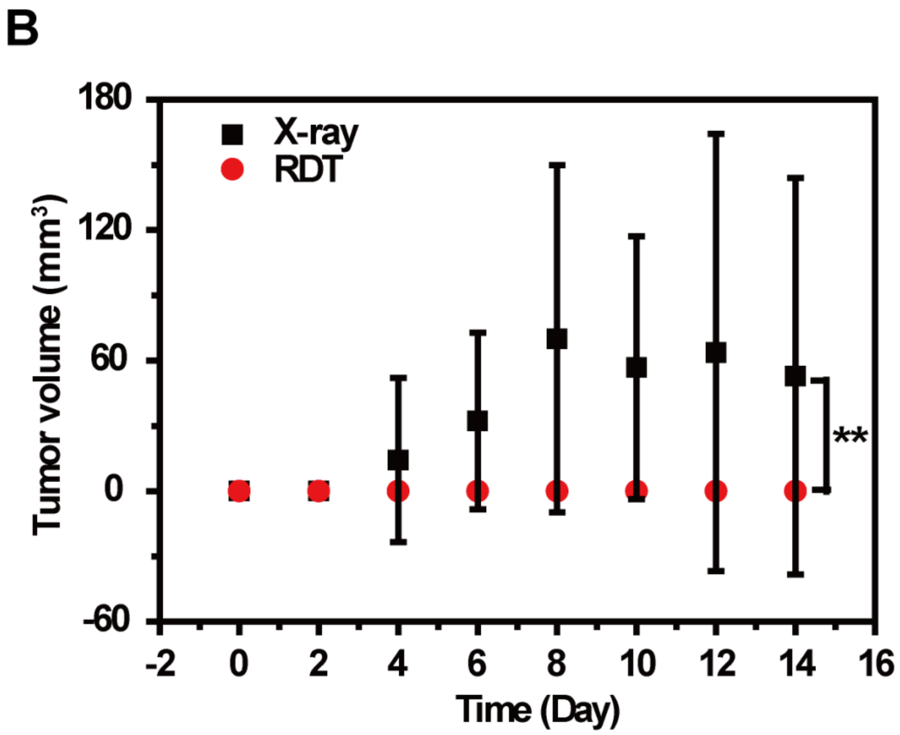

Figure S12. (A) *In vivo* AuNC@DHLA-based radiodynamic therapy. Tumor growth curve of Hepa 1-6 tumor-bearing C57BL/6j mice from different groups within 26 days.

The day of injection was designated as day 0. These mice injected only with PBS were designated as the control group. These mice irradiated with X-ray at dose of 1.0 Gy fraction^{-1} for a total of 6 fractions on consecutive days, without AuNC@DHLA, were designated as the X-ray group. These mice injected with AuNC@DHLA (6.1 mg kg^{-1}) for a total of 3 fractions every other day, without X-ray radiation, were designated as the AuNC group. These mice injected with AuNC@DHLA (6.1 mg kg^{-1}) and irradiated with X-ray at dose of 1.0 Gy were designated as the RDT group; Data are represented as mean ± SD (N ≥ 5 per group). Data were analyzed by two-tailed t-test (**p < 0.01, N.S. p > 0.05). (B) Tumor growth curve of RDT group and X-ray group mice challenged by subcutaneous injection of Hepa 1-6 cell suspension into the left flanks. The day of injection was designated as day 0. Data are represented as mean ± SD (4 mice per group). Data were analyzed by two-tailed t-test (**p < 0.01).

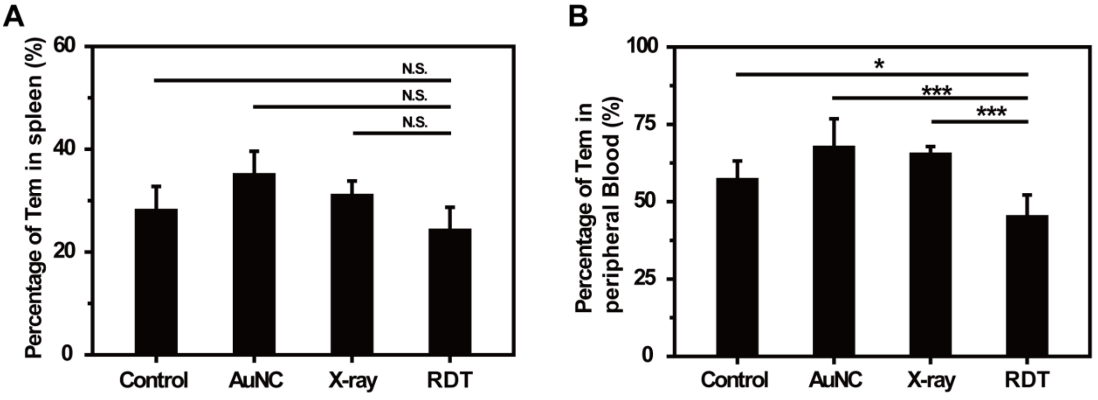

Figure S13. (A) The percentage of T effector memory cells (Tem) in spleen and (B) in peripheral blood of different groups on the 15th day after RDT treatment. Data were analyzed by two-tailed t-test (***p < 0.001, *p < 0.05, N.S. p > 0.05).

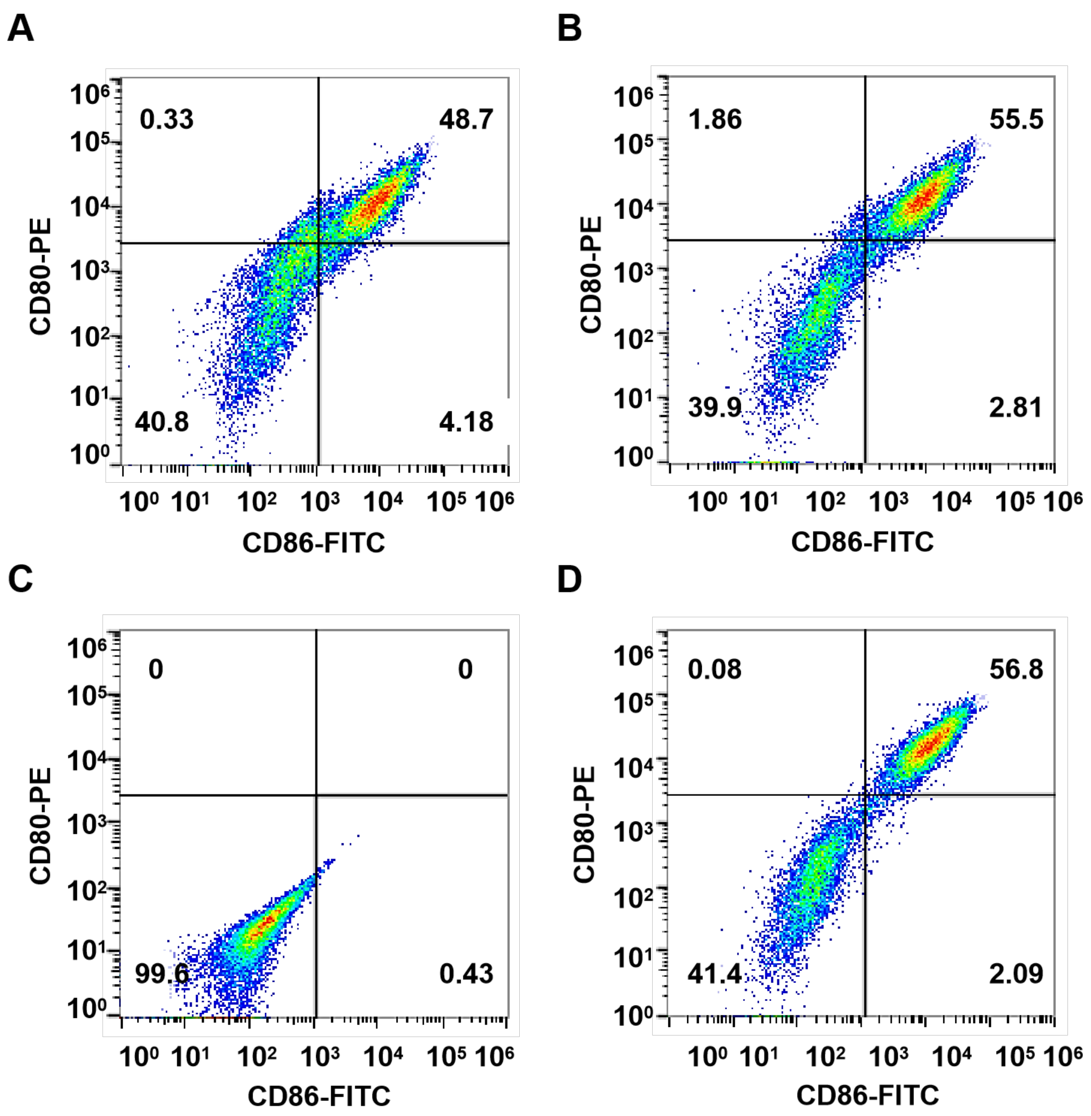

Figure S14. Activation of immature dendritic cells deriving from bone marrow of C57BL/6j mice treated without and with AuNC@DHLA. (A) Control group, (B) AuNC group, (C) Negative group and (D) Positive group with lipopolysaccharide (LPS). The concentration of AuNC@DHLA was 100 μg mL^{-1}.

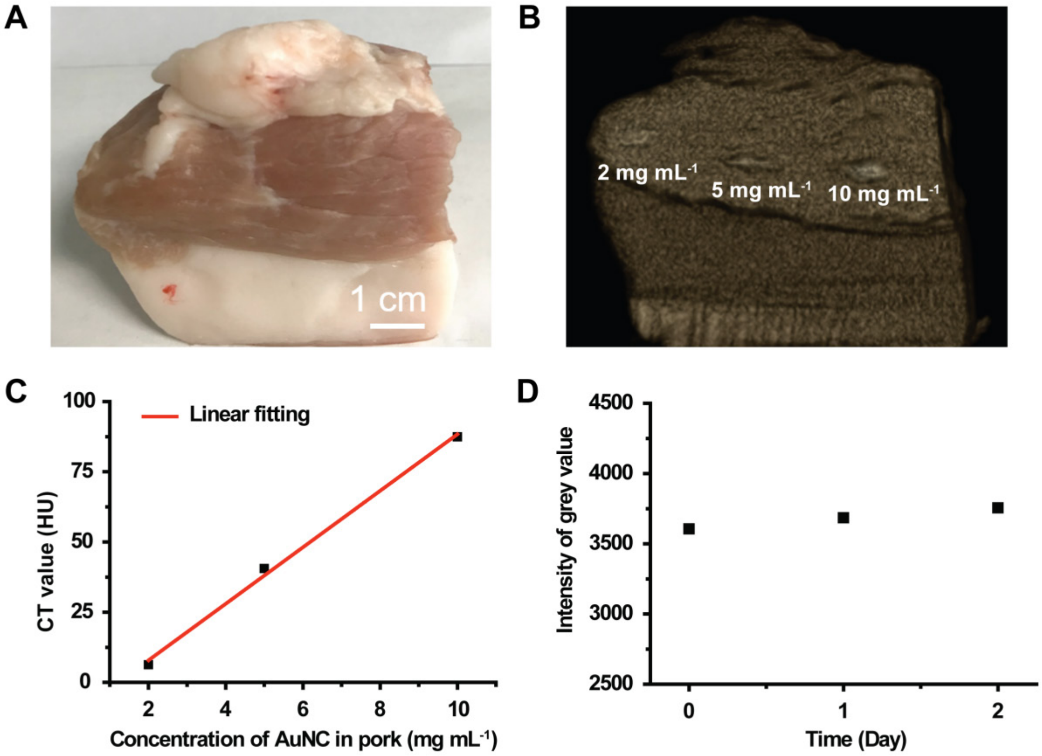

Figure S15. (A) The picture of pork. Scale bar = 1 cm. (B) CT imaging of pork with different concentration of AuNC@DHLA. (C) Intensity of grey value of AuNC@DHLA (5.0 mg mL^{-1}) as a function of concentration. The result of linear curve fitting is: Y = 10.06*X - 12.27, R^2 = 0.99. (D) Intensity of grey value of AuNC@DHLA (5.0 mg mL^{-1}) within the period of 48 h.

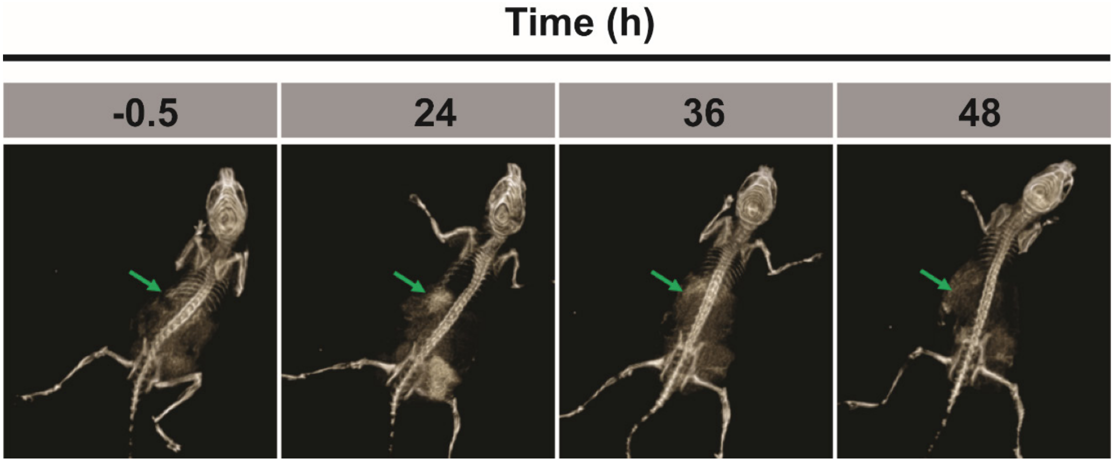

Figure S16. *In vivo* 3D CT images of the liver of mice (indicated by green arrows) at each time point. The AuNC@DHLA (10.0 mg mL^{-1}) might be cleared out of the body from kidney.

Table S1. The survival fraction (SF) of colony formation assay.

The dosage of X-ray (Gy)	SF of colony formation assay (%) in the X-ray group	SF of colony formation assay (%) in the RDT group
**0**	100 ± 4.34	94.08 ± 7.29
**0.25**	86.48 ± 5.16	53.24 ± 3.68
**0.5**	78.59 ± 1.46	46.20 ± 2.13
**1.0**	50.14 ± 6.83	25.35 ± 4.71
**2.0**	12.11 ± 1.29	3.10 ± 0.98

Table S2. The tumor growth inhibition index (TGI).

Time (Day)	TGI of AuNC group (%)	TGI of X-ray group (%)	TGI of RDT group (%)
**0**	0 ± 0.81	0 ± 1.03	0 ± 0.74
**4**	23.33 ± 0.74	0 ± 0.79	25.22 ± 0.72
**8**	10.00 ± 1.68	10 ± 0.99	44.75 ± 0.55
**12**	9.65 ± 0.62	8.27 ± 0.83	52.26 ± 0.41
**16**	11.85 ± 0.62	1.62 ± 0.73	53.79 ± 0.52
**20**	0 ± 0.60	0 ± 1.00	60.79 ± 0.75

References

1. Han R, Zhao M, Wang Z, Liu H, Zhu S, Huang L, et al. Super-efficient two-photon photodynamic therapy with a gold nanocluster as a type I photosensitizer. ACS Nano. 2020; 14: 9532-44.
2. Kim JA, Åberg C, de Cárcer G, Malumbres M, Salvati A, Dawson K A. Low dose of amino-modified nanoparticles induces cell cycle arrest. ACS Nano. 2013; 7: 7483-94.
